# Chromatin-associated degradation is defined by UBXN-3/FAF1 to safeguard DNA replication fork progression

**DOI:** 10.1038/ncomms10612

**Published:** 2016-02-04

**Authors:** André Franz, Paul A. Pirson, Domenic Pilger, Swagata Halder, Divya Achuthankutty, Hamid Kashkar, Kristijan Ramadan, Thorsten Hoppe

**Affiliations:** 1 Institute for Genetics and CECAD Research Center, University of Cologne, Joseph-Stelzmann-Str. 26, 50931 Cologne, Germany; 2 Department of Oncology, University of Oxford, Cancer Research UK/Medical Research Council Oxford, Institute for Radiation Oncology, Old Road Campus Research Building, OX3 7DQ Oxford, UK; 3 Centre for Molecular Medicine Cologne and Institute for Medical Microbiology, Immunology and Hygiene at CECAD Research Center, University Hospital of Cologne, Joseph-Stelzmann-Str. 26, 50931 Cologne, Germany

## Abstract

The coordinated activity of DNA replication factors is a highly dynamic process that involves ubiquitin-dependent regulation. In this context, the ubiquitin-directed ATPase CDC-48/p97 recently emerged as a key regulator of chromatin-associated degradation in several of the DNA metabolic pathways that assure genome integrity. However, the spatiotemporal control of distinct CDC-48/p97 substrates in the chromatin environment remained unclear. Here, we report that progression of the DNA replication fork is coordinated by UBXN-3/FAF1. UBXN-3/FAF1 binds to the licensing factor CDT-1 and additional ubiquitylated proteins, thus promoting CDC-48/p97-dependent turnover and disassembly of DNA replication factor complexes. Consequently, inactivation of UBXN-3/FAF1 stabilizes CDT-1 and CDC-45/GINS on chromatin, causing severe defects in replication fork dynamics accompanied by pronounced replication stress and eventually resulting in genome instability. Our work identifies a critical substrate selection module of CDC-48/p97 required for chromatin-associated protein degradation in both *Caenorhabditis elegans* and humans, which is relevant to oncogenesis and aging.

## Abstract (web summary)

Cdc48/p97 is a key component of the ubiquitin-proteasome system, acting as a ubiquitin-directed segregase to regulate multiple cellular functions. Here the authors identify UBXN-3/FAF1 as a crucial regulator of chromatin-associated protein degradation that recruits Cdc48/p97 to DNA replication forks.

## Graphical abstract

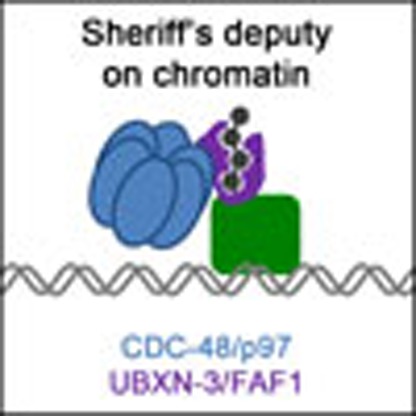

Duplication of the genomic information is a key task for dividing cells. The intricacy of DNA replication is reflected by the plethora of regulatory factors that promote different steps of DNA synthesis^1^. The assembly of DNA replication factors into specialized subcomplexes is tightly controlled to ensure genome stability. Accordingly, misregulation of DNA replication generates fatal consequences, resulting in inefficient DNA synthesis and chromosomal damage, which ultimately cause tumorigenesis or stem-cell depletion^2^,^3^,^4^,^5^,^6^.

The licensing factor CDT-1 (chromatin and DNA licensing factor 1) initiates the formation of a pre-replication complex (pre-RC) at replication origins once per cell cycle^7^,^8^. Assembled pre-RCs represent origins that are approved for DNA replication. However, pre-RCs remain passive until their activation during S phase^9^,^10^. Upon transition into DNA synthesis, the replication activation factors (cell division cycle protein 45) CDC-45 and the Go-Ichi-Nii-San (GINS) complex associate with pre-RCs^11^,^12^. GINS binding facilitates the recruitment of further replication factors including the DNA polymerases, which triggers the elongation phase of DNA replication^13^,^14^. A central factor that coordinates the described licensing and elongation events is CDC-48/p97 (Cdc48p in yeast, CDC-48 in nematodes, p97 or (Valosin containing protein) in mammals), VCP a ubiquitin-selective ATPase. Importantly, our recent findings identified that CDC-48 links ubiquitin-dependent degradation of CDT-1 to the release of the CDC-45/GINS complex in *Caenorhabditis elegans*^15^,^16^. Depletion of CDC-48 or its heterodimeric cofactor UFD-1/NPL-4 (ubiquitin fusion degradation protein 1, nuclear protein localization 4) results in stabilization of CDT-1 exclusively on mitotic chromatin. In addition, CDC-45 and the GINS subunit SLD-5 (synthetic lethal with dpb11-1 protein 5) persist on chromatin, becoming visible after S phase is completed upon CDC-48^UFD-1/NPL-4^ inactivation. Thus, CDC-48 coordinates chromatin association of different protein complexes with replication fork progression. Consequently, inhibition of CDC-48 results in severe S-phase progression defects, due to activation of the DNA replication checkpoint^15^,^16^. Work in *Xenopus laevis* and human cells identified a related requirement for CDT-1/Cdt1 mobilization and turnover, emphasizing a crucial function of CDC-48/p97 in eukaryotic DNA replication^16^,^17^.

CDC-48/p97 is a key component of the ubiquitin/proteasome system, important for mobilization and targeting of ubiquitylated substrates for degradation by the 26S proteasome^18^. Interestingly, it regulates diverse cellular processes such as degradation of proteins associated with the endoplasmic reticulum (ER-associated degradation, ERAD) or mitochondria (mitochondria-associated degradation, MAD), cell-cycle progression and lysosomal proteolysis^18^,^19^. Recently, CDC-48/p97 emerges as a central regulator of chromatin-associated degradation (CAD), which is relevant to genome stability and human genetic disorders including cancer and accelerated aging^20^,^21^,^22^,^23^. With the increasing number of CDC-48/p97 substrate proteins, it is becoming evident that additional regulatory mechanisms specifying substrate selection at a given time need to be identified. Especially, the cell-cycle-dependent coordination of distinct events during DNA replication necessitates precise spatial and temporal regulation of CDC-48 function at the chromatin^16^,^22^,^23^,^24^,^25^.

To address how CDC-48-dependent DNA replication is adjusted with substrate recruitment and cell-cycle progression, we screened for genetic interactors of *cdc-48* in *C. elegans*. Using an RNA interference (RNAi)-based approach, we identified UBXN-3 (ubiquitin regulatory X (UBX) domain-containing protein in nematodes 3) as a crucial factor that coordinates CDC-48-dependent DNA replication events. Embryos with reduced protein levels of both UBXN-3 and CDC-48 recapitulate phenotypes caused by complete depletion of CDC-48, including the accumulation of CDT-1 and GINS on mitotic chromatin. UBXN-3 binds to CDT-1, thus facilitating CDC-48 recruitment for substrate turnover and disassembly at the chromatin. Consequently, depletion of the human homologue FAS-associated factor 1 (FAF1) results in stabilization of CDT-1 protein, pronounced decrease in replication fork velocity, increased number of stalled forks, and newly fired origins, cumulating in the activation of the DNA damage response. In conclusion, our results demonstrate an evolutionarily conserved role of UBXN-3/FAF1 in orchestrating CAD of DNA replication factors to safeguard genome integrity, which is relevant to the emerging link between FAF1 deletion and cancer development^26^ (and references therein).

## Results

### Embryonic survival depends on UBXN-3 function

**Figure 1:**
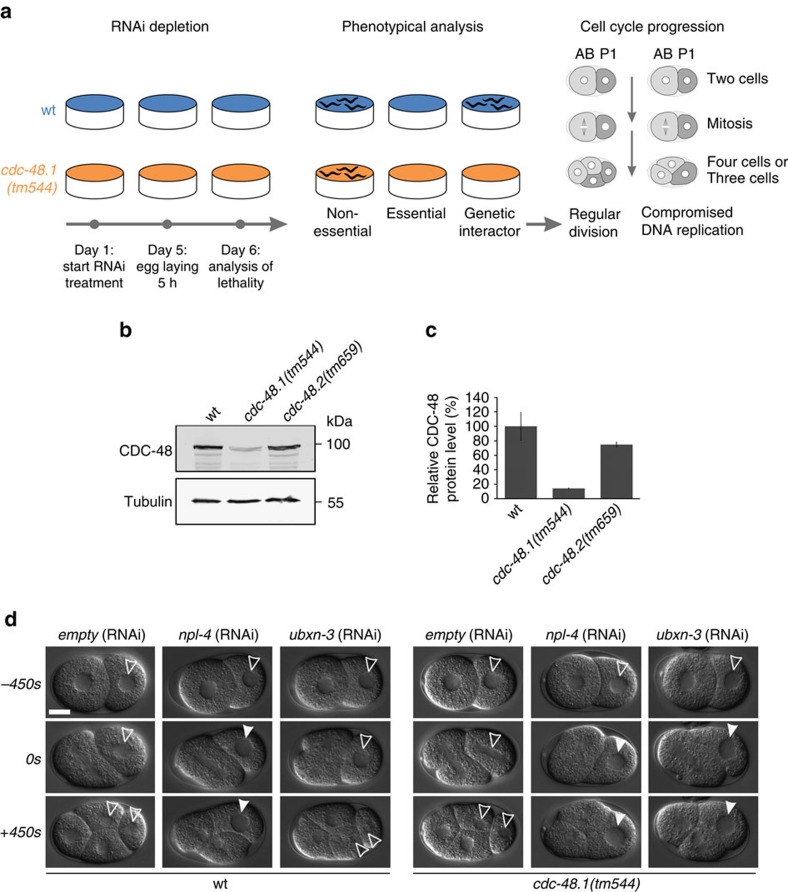
Worms lacking CDC-48.1 are sensitized for *ubxn-3* depletion. (**a**) Schematic illustration of the RNAi screening procedure to identify genetic interactors of *cdc-48.1*. Wild-type (wt) worms (blue) and *cdc-48.1(tm544)* deletion mutants (orange) were depleted for candidate genes by RNAi before analysis of embryonic lethality or developmental growth defects (indicated by an empty plate). Potential interactor genes were analysed for cell division defects by time-lapse microscopy. (**b**,**c**) Western blot analysis of CDC-48 and tubulin protein levels in embryonic extracts of wt, *cdc-48.1* or *cdc-48.2* deletion mutants. Graph shows CDC-48 level relative to tubulin. Error bars show s.d. of mean values generated in five independent experiments. (**d**) Selected pictures of time-lapse recordings of wt or *cdc-48.1(tm544)* embryos depleted for indicated genes by RNAi. To visualize a prolonged three-cell stage, embryonic division is shown at onset of mitosis in the AB cell (0 s) and respective time points before or thereafter (±450 s). Empty arrowheads indicate wild-type-like division pattern; filled arrowheads indicate delayed cell division of the P1 cell. Scale bar, 10 μm.

The ubiquitin-selective segregase CDC-48/p97 is a key regulator of numerous protein degradation pathways within eukaryotic cells^18^,^19^. Besides substrate turnover in different cellular compartments, we and others identified a central role of CDC-48/p97 in DNA metabolic pathways that safeguard genome stability^20^,^21^,^22^,^23^. This complexity of degradation pathways demands mechanisms that accurately coordinate CDC-48/p97 activity. To identify factors that provide substrate specificity and spatiotemporal regulation of CDC-48 during DNA replication, we performed an RNAi-based candidate screen targeting potential interactors (Fig. 1a; Supplementary Table 1). A set of 137 RNAi clones was used for depletion of 95 genes, followed by microscopic evaluation of developmental phenotypes and cell-cycle progression defects (Fig. 1a,d; Supplementary Table 1). In addition to wild-type (wt) nematodes, downregulation of candidate genes was also performed in viable *cdc-48.1(tm544)* deletion mutants, representing a sensitized background with a reduction of 80% in total CDC-48 protein levels in embryos (Fig. 1b,c). The remaining 20% of CDC-48 protein are encoded by the *cdc-48.2* gene, which provides its essential function under untreated conditions. In fact, the screen identified several genes required for development and viability when depleted in the wt or the *cdc-48.1(tm544)* mutant (Supplementary Fig. 1e; Supplementary Table 1).

We have previously shown that CDC-48^UFD-1/NPL-4^-depleted embryos exhibit a pronounced delay in S-phase progression caused by activation of the conserved DNA replication checkpoint kinases ATL-1/ATR and CHK-1/Chk1 (refs 15, 27). Among all genetic interactors identified in our candidate approach, embryos specifically lacking UBXN-3 in addition to CDC-48.1 exhibited a characteristic cell division phenotype reminiscent of complete CDC-48 depletion or loss of its cofactors UFD-1/NPL-4 (refs 15, 16; Fig. 1d and Supplementary Fig. 1f). Indeed, time-lapse microscopy verified a prolonged three-cell stage of *cdc-48.1(tm544); ubxn-3(RNAi)* embryos, which is indicative of compromised DNA replication^27^,^28^ (Fig. 1a,d). Accordingly, UBXN-3 localizes inside the nucleus during S phase, comparable to CDC-48 (Supplementary Fig. 1a,g)^15^. Western blot analysis confirmed unchanged amounts of CDC-48 and UFD-1/NPL-4 proteins in *ubxn-3*-deletion mutants, excluding indirect effects on CDC-48^UFD-1/NPL-4^ complex integrity (Supplementary Fig. 1b–d). Thus, our candidate approach identified *ubxn-3* as a potent genetic interactor of *cdc-48.1* important for cell-cycle progression.

### UBXN-3 promotes DNA replication and genome stability

**Figure 2:**
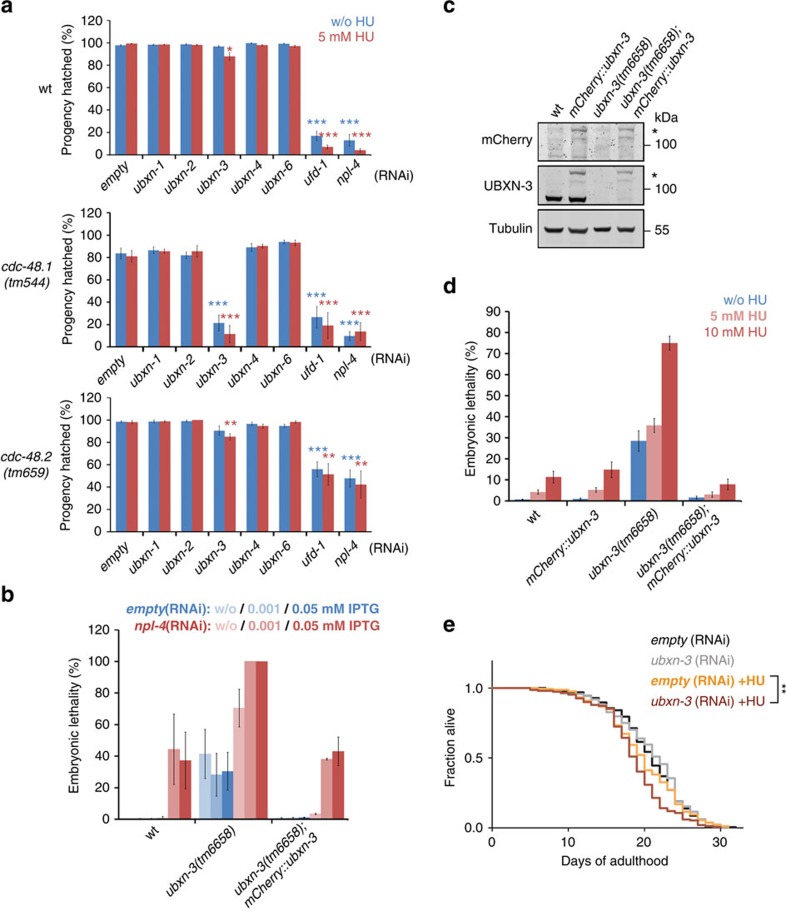
Depletion of UBXN-3 results in DNA replication stress. (**a**) Developmental defects of worms RNAi depleted for indicated genes in respective genetic backgrounds exposed to 5 mM hydroxyurea (HU). Mean values were calculated from eight independent replicates for wt and *cdc-48.1(tm544)*, or four replicates for *cdc-48.2(tm659)*. Error bars show s.e.m. Statistical significance refers to *empty* (RNAi) conditions without HU (blue asterisks), or with 5 mM HU (red asterisks), respectively. (**b**) *npl-4(RNAi)* sensitivity of worms with indicated genotypes. Error bars show s.e.m. Mean values were calculated from four independent replicates. (**c**) Western blot analysis of mCherry, UBXN-3, and Tubulin protein levels in synchronized adult worms of indicated genotypes. Asterisk on the right marks the mCherry::UBXN-3 fusion protein. (**d**) Sensitivity of worms with indicated genotypes towards distinct HU concentrations. Graph shows mean embryonic lethality of six replicates. Error bars show s.e.m. (**e**) Adult lifespan after RNAi depletion of control (*empty*) or *ubxn-3* with or without 10 mM HU. Data were collected from five replicates, in sum representing mean lifespan of at least 165 individuals per condition. In (**a**) the single asterisk indicates a *P* value of ≤0.05, the double asterisk ≤0.001 and the triple asterisk indicates *P* values of ≤0.0001 in Student's *t*-test. In (**e**) the double asterisk indicates a *P* value of ≤0.01 in Mantel–Cox test.

Since the cell-cycle progression phenotype suggested a close cooperation between CDC-48 and UBXN-3 in DNA replication (Fig. 1d)^15^,^16^, we monitored sensitivity of wt and *cdc-48.1(tm544)* mutant embryos lacking UBXN-3 towards the DNA replication inhibitor hydroxyurea (HU)^29^. Strikingly, codepletion of UBXN-3 and CDC-48.1 strongly impaired embryonic viability, which further aggravated in the presence of HU, thus phenocopying *ufd-1* and *npl-4* RNAi defects (Fig. 2a). The effect of UBXN-3 depletion is highly specific since downregulation of genes encoding the related UBX proteins UBXN-1, UBXN-2, UBXN-4 and UBXN-6, neither enhanced HU sensitivity nor resulted in synthetic phenotypes (Fig. 2a; Supplementary Fig. 2c). Both, embryonic defects and HU sensitivity of UBXN-3 depletion strictly correlate with the amount of CDC-48 protein. Consequently, *ubxn-3(RNAi)* caused significant but less pronounced HU sensitivity in *cdc-48.2(tm659)* mutants, lacking only 20% of the overall CDC-48 protein level, as compared with the *cdc-48.1(tm544)* deletion (Fig. 2a). This genetic interaction supports the idea that CDC-48 and UBXN-3 together facilitate DNA replication and cell-cycle progression. Likewise, the *ubxn-3(tm6658)* deletion mutant is hypersensitive towards *npl-4(RNAi)* conditions that do not markedly affect embryonic survival in the wt (Fig. 2b). In line with this genetic epistasis, UBXN-3 and NPL-4 have been shown to simultaneously bind to single CDC-48 hexamers *in vivo*^30^. These data support the idea that UBXN-3 functionally cooperates with CDC-48^UFD-1/NPL-4^ during embryogenesis.

**Figure 3:**
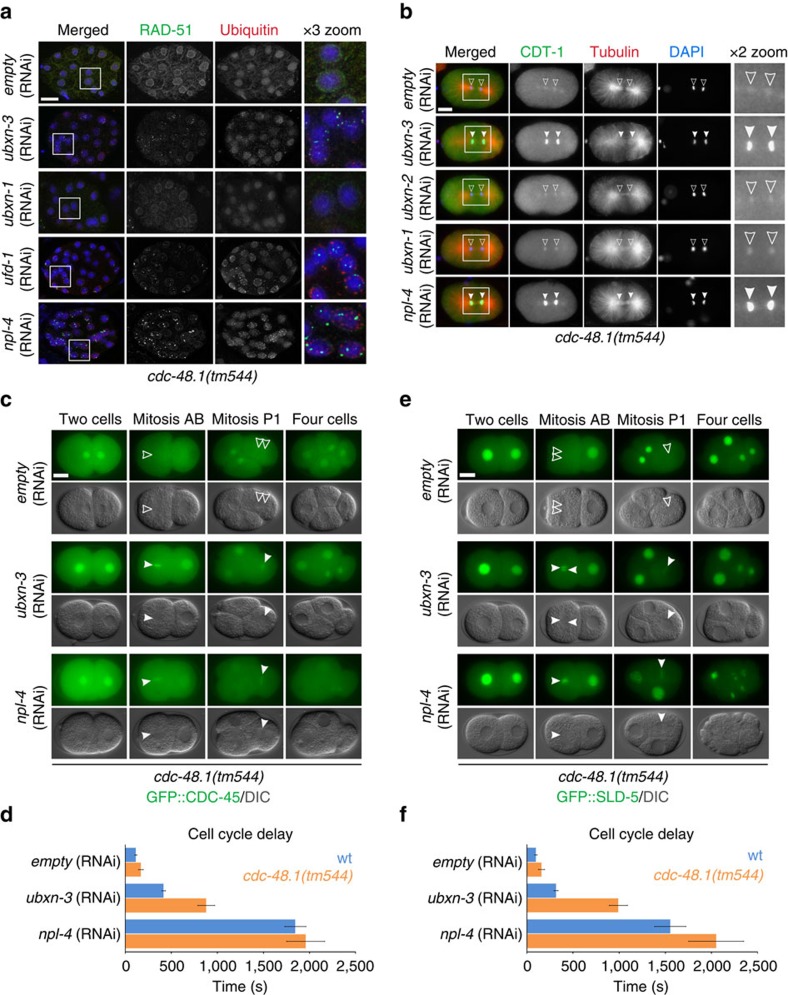
CDT-1 and CDC-45/GINS accumulate on mitotic chromatin in embryos lacking UBXN-3 and CDC-48.1. (**a**) Representative projections of confocal z-stacks of embryos RNAi depleted for indicated genes in *cdc-48.1(tm544)* mutant. Embryos were immunostained for RAD-51 (green) and conjugated ubiquitin (FK2) (red). DNA was stained with DAPI (blue). The boxed area is three times magnified (× 3 zoom). Scale bar, 10 μm. (**b**) Immunostainings of one-cell stage *cdc-48.1(tm544)* mutant embryos RNAi depleted for indicated genes. CDT-1 (green), tubulin (red) and DAPI (blue) staining is shown as merge images and in separate channels. The boxed area is magnified by two times (× 2 zoom). Empty arrowheads indicate wt-like CDT-1 levels, filled arrowheads highlight increased CDT-1 levels on anaphase chromatin. Scale bar, 10 μm. (**c**,**e**) Selected images of time-lapse recordings of *cdc-48.1(tm544)* embryos expressing GFP::CDC-45 (green) or GFP::SLD-5 (green) that are depleted for *empty* control, *ubxn-3* and *npl-4*. Each image series shows representative cell-cycle phases of the mitotic division in the AB and P1 cell, respectively. Empty arrowheads point to wild-type-like CDC-45 and SLD-5 localization, filled arrowheads indicate persistent association of the indicated proteins with mitotic chromatin. DIC is shown in grey. Scale bar, 10 μm. (**d**,**f**) Quantification of the cell delay between AB and P1 cell division (cell-cycle delay) of wt (blue) and *cdc-48.1(tm544)* (orange) embryos depleted for *empty*, *ubxn-3* and *npl-4* by RNAi. Time is shown in seconds. Evaluation of cell division timing was done in embryos expressing GFP::CDC-45 (**c**) and GFP::SLD-5 (**e**), respectively. Error bars show s.e.m. Mean values are based on two to six individual recordings per condition.

Functional analysis of the *ubxn-3(tm6658)* deletion mutant further supports our observation that UBXN-3 is required for embryonic survival, particularly when DNA replication is compromised upon exposure to HU (Fig. 2d). In addition, significant HU sensitivity was also observed upon *ubxn-3* knockdown by RNAi (Supplementary Fig. 2c). Strikingly, germline-specific expression of mCherry::UBXN-3 restores the basal 30% embryonic lethality of the *ubxn-3(tm6658)* allele (Fig. 2b–d). Furthermore, transgenic expression of UBXN-3 in the *ubxn-3(tm6658)* deletion background complements sensitivity towards *npl-4(RNAi)* as well as HU (Fig. 2b,d). To provide additional evidence for DNA replication defects, we assessed the accumulation of RAD-51 recombinase on DNA. Similar to embryos lacking UFD-1/NPL-4, codepletion of UBXN-3 and CDC-48.1 resulted in formation of characteristic RAD-51 foci, indicating the formation of DNA double-strand breaks upon replication fork collapse^31^,^32^ (Fig. 3a; Supplementary Fig. 3a). Besides embryonic defects, HU treatment of young adult worms depleted for UBXN-3 resulted in reduced lifespan, implicating genome instability and premature aging (Fig. 2e). In conclusion, UBXN-3 is critical for embryonic development and compensation of DNA replication stress.

CDC-48 function is required for protein degradation in distinct cellular compartments, such as the unfolded protein response in the ER or mitochondria. The expression levels of HSP-4 or HSP-6 chaperones directly monitor defects in degradation of misfolded proteins accumulating in the ER lumen or at mitochondria^33^,^34^. Importantly, neither HSP-4 nor HSP-6 expression were induced after *ubxn-3(RNAi)* (Supplementary Fig. 2a,b). In conclusion, UBXN-3 provides a highly specific role in DNA metabolism.

### CDC-48^UBXN-3^ governs chromatin-bound replication factors

Our recent findings identified that CDC-48 works together with the canonical cofactor UFD-1/NPL-4 in degradation and disassembly of chromatin-associated DNA replication factors^16^,^17^. Consequently, inactivation of the CDC-48^UFD-1/NPL-4^ complex causes increased levels of the DNA licensing factor CDT-1 on mitotic chromosomes as well as persistent chromatin association of CDC-45 and SLD-5, a subunit of the tetrameric GINS complex^16^ (Fig. 3b,c,e; Supplementary Fig. 3b,c). In embryonic cell cycles, the loading of DNA replication licensing factors is initiated when cells are morphologically engaged in mitosis^35^. Consequently, we use the description mitotic CDT-1 stabilization to clearly discriminate between these early licensing events and apparently distinct CDT-1 regulatory mechanisms at later stages^16^. Given the identification of *ubxn-3* as a genetic interactor of *cdc-48* and *npl-4* important for DNA replication and genome stability, we next addressed whether UBXN-3 is involved in the regulation of these CDC-48 targets. Strikingly, depletion of UBXN-3 in the *cdc-48.1(tm544)* mutant resulted in elevated levels of CDT-1 on mitotic chromatin as revealed by immunostaining (Fig. 3b). In contrast, *ubxn-1* or *ubxn-2* (RNAi) did not interfere with CDT-1 regulation in the absence of CDC-48.1 (Fig. 3b; Supplementary Fig. 3b). During unperturbed cell divisions, localization of the replisome components CDC-45 and GINS is restricted to S-phase nuclei^12^,^16^,^35^. Remarkably, green fluorescent protein (GFP) fusions of CDC-45 and SLD-5 persist on the chromatin upon UBXN-3 and CDC-48.1 codepletion, becoming visible on condensed mitotic chromosomes (Fig. 3c,e; Supplementary Fig. 3c,d). As a consequence, genotoxic stress manifests in a pronounced delay in cell-cycle progression following UBXN-3 depletion in the wt and *cdc-48.1(tm544)* background (Fig. 3d,f; Supplementary Fig. 3e). This cell-cycle delay phenotype results from activation of the DNA replication checkpoint, which can be visualized by measurement of cell division timing (Fig. 1a)^27^,^28^.

These findings suggest that UBXN-3 cooperates with CDC-48 in DNA replication, including degradation of CDT-1 and disassembly of CDC-45/GINS at the chromatin. Consequently, UBXN-3 depletion provokes DNA replication stress, which is reflected by delayed cell-cycle progression due to activation of the intra-S-phase checkpoint.

### UBXN-3 and CDC-48 form a functional complex in the nucleus

**Figure 4:**
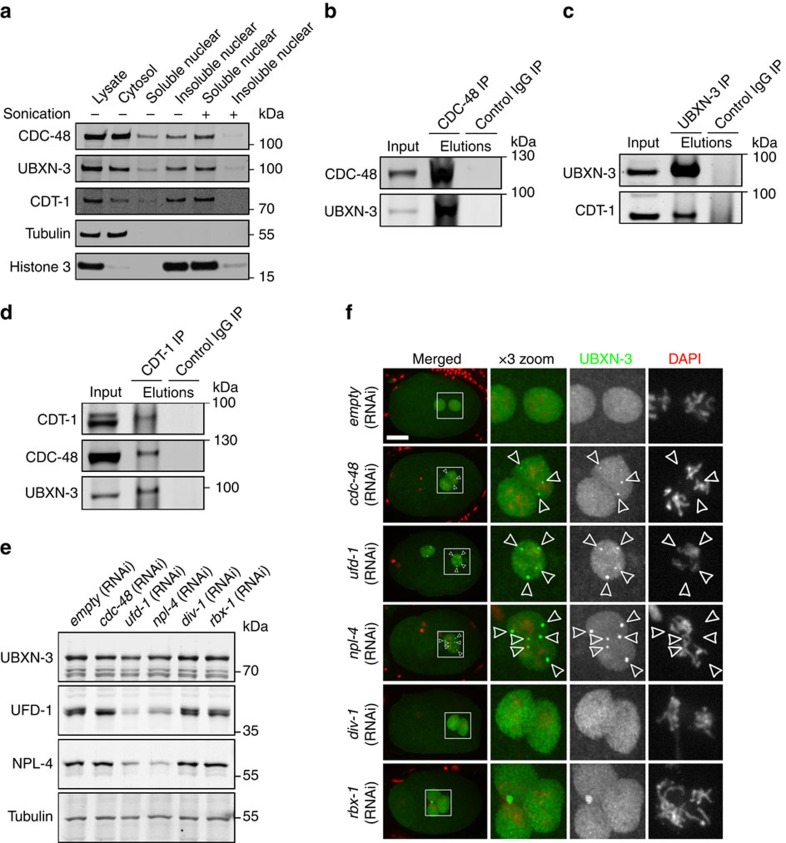
UBXN-3 interacts with CDC-48 and CDT-1 *in vivo*. (**a**) Cellular fractionation of *C. elegans* embryos analysed by western blotting to detect CDC-48, UBXN-3, CDT-1, tubulin and histone 3, respectively. Nuclear fractions were left untreated or sonicated to release associated proteins from the chromatin. (**b**–**d**) wt worm lysates were incubated with the indicated antibodies coupled to Dynabeads Protein A for immunoprecipitation and subsequently analysed by western blotting for CDC-48, UBXN-3 and CDT-1. (**e**) Western blot analysis of embryonic lysates depleted for indicated genes by RNAi. (**f**) Projections of confocal images of one-cell stage embryos depleted for indicated genes by RNAi. Embryos were immunostained for UBXN-3 (green). DNA was stained with DAPI (red). The boxed area is magnified by three times (× 3 zoom). Arrowheads indicate nuclear punctae of UBXN-3. Scale bar, 10 μm.

Given its functional relevance in DNA replication, we tested whether UBXN-3 is physically linked to chromatin. Indeed, cellular fractionation of *C. elegans* embryonic lysates confirmed high abundance of UBXN-3, CDC-48 and CDT-1 in purified nuclei (Fig. 4a). Upon disruption of DNA polymers by sonication chromatin-associated proteins relocalized into the soluble nuclear fraction, implicating that UBXN-3 and CDC-48 are associated with the chromatin similar to CDT-1 (Fig. 4a).

To address whether UBXN-3 also physically interacts with CDC-48, we performed co-immunoprecipitation (co-IP) experiments using *C. elegans*-specific antibodies on whole-worm lysates followed by western blot analysis. Importantly, endogenous UBXN-3 protein co-purified with CDC-48 (Fig. 4b). Furthermore, UBXN-3 is also able to bind the CDC-48 substrate CDT-1 when precipitated from worm lysate (Fig. 4c; Supplementary Fig. 4a). Co-IP experiments targeting CDT-1 as an antigen even revealed simultaneous interactions among CDT-1, CDC-48 and UBXN-3 (Fig. 4d). Taken together, these results suggest the existence of a CDC-48^UBXN-3^ complex that mediates CDT-1 recruitment *in vivo*.

On the basis of these protein–protein interactions, we wondered whether the nuclear localization of UBXN-3 depends on integrity of the CDC-48^UFD-1/NPL-4^ complex (Supplementary Fig. 1a). Indeed, RNAi-mediated depletion of CDC-48 or the cofactor UFD-1/NPL-4 resulted in the formation of UBXN-3-positive punctae in the nucleoplasm of worm embryos (Fig. 4f). In contrast to the UFD-1/NPL-4 dimer, however, UBXN-3 protein levels remained stable upon downregulation of the CDC-48^UFD-1/NPL-4^ complex^15^,^16^,^36^ (Fig. 4e,f). Depletion of the DNA polymerase subunit DIV-1, known to cause replication stress and checkpoint activation, did not result in UBXN-3 mislocalization^28^ (Fig. 4e,f). Likewise, *rbx-1(RNAi)*, which stabilizes CDT-1 specifically during S phase and consequently causes genomic instability, resulted in formation of UBXN-3-positive micronuclei rather than the characteristic UBXN-3 punctae inside the nucleus^16^,^37^ (Fig. 4e,f). Thus, proper nuclear localization of UBXN-3 during S phase strictly depends on a functional CDC-48^UFD-1/NPL-4^ complex.

### UBXN-3 binds to CDC-48-dependent substrate proteins

**Figure 5:**
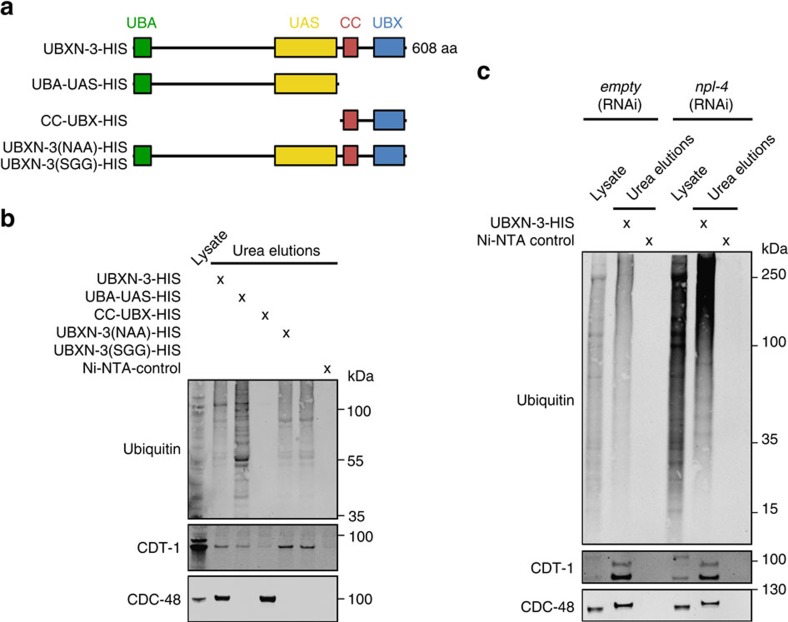
Functional analysis of UBXN-3-binding domains. (**a**) Schematic illustration of full-length UBXN-3, truncated peptides and FPK-motif mutants used for binding studies. Full-length UBXN-3b consists of 608 amino acids (aa). (**b**,**c**) wt or NPL-4-depleted worm lysates were incubated with the indicated, recombinant UBXN-3 variants coupled to Ni-NTA beads and analysed by western blotting. Eluted proteins were detected by immunolabeling of ubiquitin conjugates (FK2), CDT-1 and CDC-48.

UBXN-3 belongs to a subclass of cofactors that share the UBX domain as a characteristic CDC-48-binding motif^38^,^39^. Besides the UBX domain, UBXN-3 harbours a coiled coil (CC) motif, a ubiquitin-associated domain (UBA) and a UAS domain of unknown function^40^ (Fig. 5a). Furthermore, the mammalian ortholog FAF1 contains two ubiquitin-like (UBL) domains. To address the requirement of the different domains, we established an *in vitro* binding assay using purified full-length or truncated variants of UBXN-3 (Fig. 5a). Moreover, the conserved FPK/R motif in the UBX domain was point mutated to analyse CDC-48 interaction^41^ (Fig. 5a; Supplementary Fig. 5a). His-tagged UBXN-3 proteins were coupled to Ni-NTA agarose and incubated with whole-worm lysate. Associated proteins were subsequently eluted with 8 M urea buffer followed by elution of His-tagged proteins in Laemmli buffer (Fig. 5a; Supplementary Fig. 5b). Importantly, full-length UBXN-3 co-purified with CDC-48 and CDT-1 (Fig. 5b,c), thus confirming the *in vivo* results shown in Fig. 4. In contrast, a truncated version lacking the UBX and CC domain did not interact with CDC-48, however, was still able to bind CDT-1 and ubiquitin conjugates (Fig. 5b). Conversely, a fragment only consisting of the UBX and CC domain exclusively interacted with CDC-48, indicating that CDC-48 alone is not sufficient for CDT-1 binding (Fig. 5b). Remarkably, point mutations of the FPK motif in the UBX domain were completely impaired in CDC-48 interaction^41^; however, fully functional in CDT-1 and ubiquitin-conjugate binding (Fig. 5b; Supplementary Fig. 5a). The interaction with CDT-1 and CDC-48 was not markedly reduced upon *npl-4(RNAi)*, suggesting that the substrate-binding capacity of UBXN-3 is independent of the UFD-1/NPL-4 cofactor (Fig. 5c; Supplementary Fig. 5c).

These interaction studies provide evidence that UBXN-3 binds the replication factor CDT-1 and other ubiquitylated substrate proteins. Intriguingly, substrate binding occurs independently of CDC-48, suggesting a direct role for UBXN-3 in substrate recruitment.

### FAF1 is the homologue of UBXN-3 essential for DNA replication

**Figure 6:**
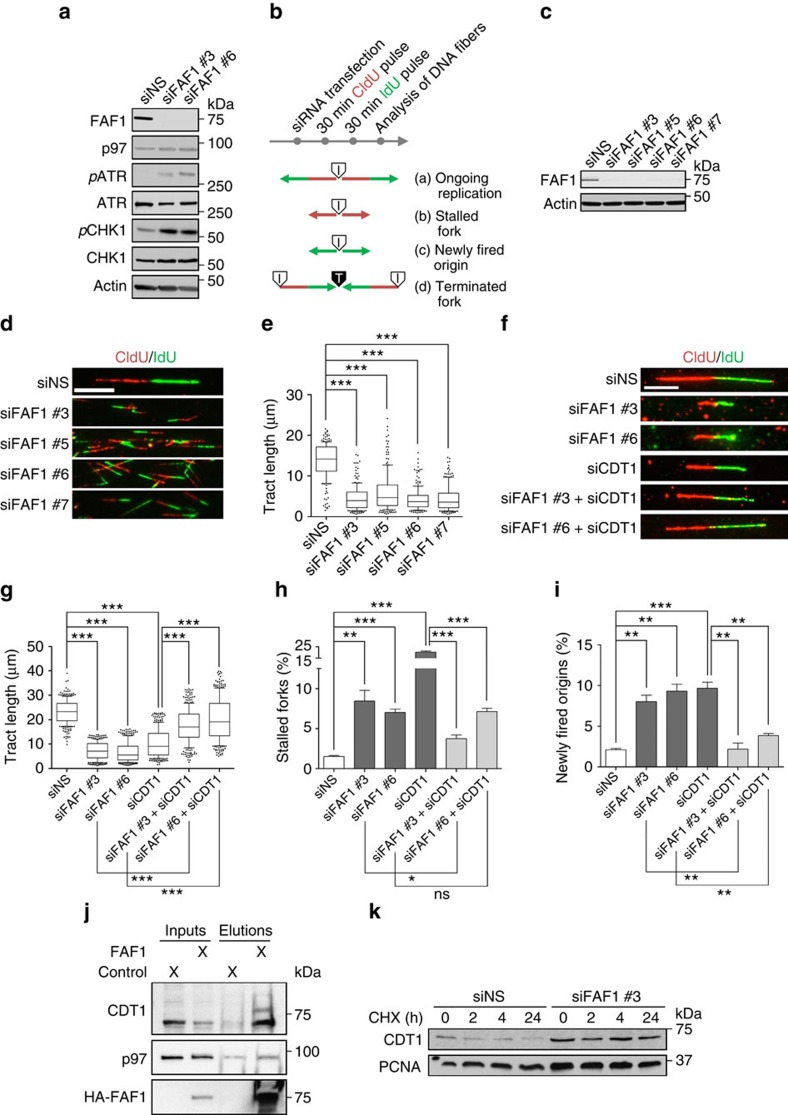
Regulation of DNA replication is conserved and FAF1-dependent in human cells. (**a**) Western blot analysis of replication checkpoint activation by phosho-specific antibodies in HEK293T cells transfected with indicated siRNAs. (**b**) Schematic illustration of DNA replication fibre analysis—(a) Indicates ongoing replication (red and green arrows), (b) stalled replication forks (red arrows only), (c) newly fired origins (green arrows only) and (d) terminated/converged forks. Sites of DNA replication initiation (I) or termination (T) are labelled, respectively. (**c**) Western blot analysis of T24 cell lysates transfected with indicated siRNAs. Efficiency of siRNA-mediated depletion is shown for FAF1 protein levels. (**d**,**e**) Representative images of microscopic analysis of DNA replication fibres by molecular combing in T24 cells after indicated siRNA transfection. CldU (first pulse) incorporation is shown in red, incorporated IdU (second pulse) in green. Quantification of replicated DNA tract length after indicated siRNA transfection in T24 cells. Tract length was determined for 100 individual forks per condition and experiment. The experiment was repeated twice. Whisker box plots show mean values and data within the 10–90 percentile. (**f**–**i**) Representative images of microscopic analysis of DNA replication fibres by molecular combing in U2OS cells after indicated siRNA transfection. Quantification of replicated DNA tract length, stalled replication forks and newly fired dormant origins after indicated siRNA transfection in U2OS cells. Tract length was determined for 100 forks per condition and experiment. Fork stalling and origin firing was quantified for 400 forks per condition and experiment. The experiment was repeated in three replicates. (**j**) Western blot analysis of control HA and HA-FAF1 co-IPs. Nuclear lysates were prepared from HEK cells expressing the p97-E587Q variant. (**k**) HEK cells were depleted for control or FAF1 by siRNA. CDT-1 and PCNA levels were monitored at indicated time points after CHX treatment by western blot analysis. Scale bar, 5 μm. Data show mean values. Error bars represent s.d.. The single asterisk indicates a *P* value of ≤0.05, the double asterisk ≤0.001 and the triple asterisk indicate *P* values of ≤0.0001.

UBXN-3 is highly conserved in metazoans and its human ortholog is termed FAF1. Notably, various *FAF1* mutations have been linked to cancer development; however, a mechanistic role in maintenance of genome stability has not been demonstrated. Therefore, we addressed whether FAF1 is also required to coordinate DNA replication in human cells. In fact, depletion of FAF1 by siRNA resulted in robust activation of the intra-S-phase checkpoint kinases ATR and CHK-1 (Fig. 6a). Moreover, FAF1 depletion caused induction of the DNA damage response, reflected by phosphorylation of ATM and CHK2 DNA repair kinases, as well as histone H2AX (Supplementary Fig. 6a). These observations implicate a conserved functional role of UBXN-3/FAF1 in DNA replication (Figs 2, 3 and 6).

To directly assess the role of FAF1 in DNA replication, we analysed the dynamics of individual replication forks by DNA fibre assays. To this end, human osteosarcoma (U2OS), urinary bladder cancer (T24) or kidney embryonic (HEK293) cells were successively pulse labelled with the nucleoside analogues 5-chloro-2′-deoxyuridine (CldU) and 5-iodo-2′-deoxyuridine (IdU), respectively, and progression of discrete replisomes was visualized by anti-CldU and anti-IdU immunofluorescence (Fig. 6b). Ongoing replication forks are labelled as tracks that contain CldU (red) followed by IdU (green), and measurement of the length of CldU/IdU tracks allows the analysis of replication fork velocity (Fig. 6b). Notably, the analysis of DNA tract length and fork velocity is exclusively based on measurement of symmetric tracts, displaying no statistical differences in the lengths of respective CldU- and IdU-single tracts^22^. In addition, this method enables visualization of stalled replication forks (CldU incorporation only) and firing of dormant origins (IdU incorporation only) as well as fork termination events (Fig. 6b). Strikingly, FAF1 depletion by different siRNAs causes pronounced DNA replication stress phenotypes in several human cell lines (Fig. 6c–i; Supplementary Fig. 6b–h). Quantification of tract lengths indicates severely defective DNA replication fork progression (Fig. 6e,g; Supplementary Fig. 6d,g), accompanied with high rates of replication fork stalling (Fig. 6h), excessive firing of dormant origins (Fig. 6i) and activation of replication stress checkpoint signalling (Fig. 6a). Notably, defects in replication fork progression upon FAF1 depletion can be completely restored upon expression of siRNA-resistant HA-FAF1 (Supplementary Fig. 6f–h), precluding any off-target effects. To exclude that the detected replication defects are secondary to inherited chromosomal aberrations or checkpoint activation of a previous cell cycle, these experiments were repeated using G0/G1-arrested T24 cells, which did not proliferate during siRNA treatment. After T24 cells were released into S phase, we monitored DNA replication forks by DNA fibre assays. In fact, several independent FAF1 siRNAs recapitulated fork progression defects similar to U2OS and HEK293 cells, reflecting direct problems in DNA synthesis during early steps of chromosomal replication (Fig. 6c–e). Although an individual depletion of either FAF1 or CDT-1 causes severe replication problems, simultaneous depletion of FAF1 together with CDT-1 clearly suppressed impaired fork dynamics (Fig. 6f–i). This finding implicates that the regulation of CDT-1 protein levels is the primary role of FAF1 during DNA replication. Altogether, these results point towards a functionally conserved role of UBXN-3/FAF1 in eukaryotic DNA replication (Figs 3b and 6f–i; Supplementary Fig. 6b–d)^16^.

**Figure 7:**
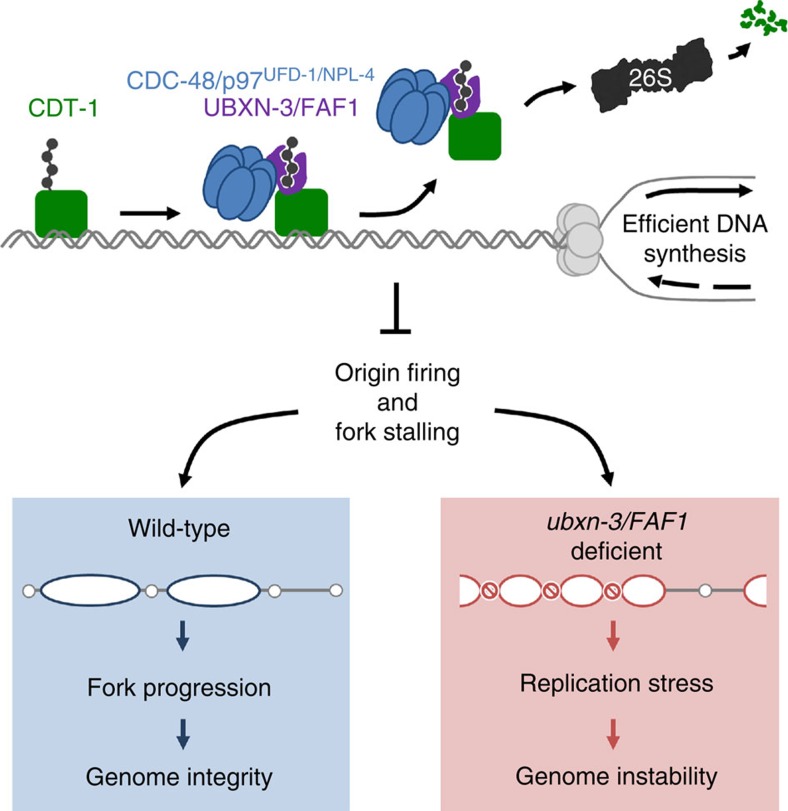
UBXN-3/FAF1 defines chromatin-associated degradation to assure DNA replication fork progression. Hypothetical model for the role of UBXN-3/FAF1 in CDC-48/p97-regulated DNA replication. UBXN-3/FAF1 (purple) binds ubiquitylated CDT-1 (green) bound to the chromatin and recruits the CDC-48/p97^UFD-1/NPL-4^ complex (blue) for disassembly and subsequent turnover of the replication factor by the 26S proteasome. Regulation of chromatin-associated CDT-1 is vital to assure efficient DNA synthesis, protecting cells from DNA replication stress.

To show that CDT-1 is a substrate of FAF1 in human cells, we performed co-IP experiments using HA-FAF1 as bait. Nuclear lysates were prepared from HEK cells expressing the p97-E587Q substrate trap variant^42^. In fact, HA-FAF1 co-purifies together with CDT-1 and p97 (Fig. 6j). To validate that FAF1 indeed determines CDT-1 turnover, we analysed protein levels in cycloheximide (CHX)-chase experiments. Strikingly, CHX treatment demonstrates that depletion of FAF1 attenuates CDT-1 degradation, resulting in stabilized protein levels (Fig. 6k). This suggests that UBXN-3/FAF1 recognizes CDT-1 as a substrate to promote p97-dependent proteasomal targeting (Fig. 7a). In summary, FAF1 plays a conserved role in DNA duplication and ensures genome stability.

## Discussion

Research during the past two decades led to the discovery of prominent cellular pathways regulated by the ubiquitin-selective ATPase CDC-48/p97 (refs 18, 19). Obviously, the increasing number of substrate proteins demands highly selective recruitment mechanisms. Given the recently discovered role of CDC-48/p97 in DNA replication, we were especially interested how CAD is coordinated to guarantee efficient DNA synthesis. As DNA replication is a fundamental cellular process that ensures the faithful propagation of the genomic information in each cell division cycle, our results are relevant to human genetic disorders including oncogenesis and premature aging syndromes^22^.

Using a genetic screening approach, we succeeded in finding a conserved UBX domain protein that provides a substrate selection module, triggering CDC-48/p97-dependent degradation at the chromatin. UBXN-3/FAF1 binds both the licensing factor CDT-1 and CDC-48/p97, and thus safeguards the timely regulation of target recognition at the replication fork. Replication stress emerging upon UBXN-3/FAF1 depletion provokes replication fork collapse and occurrence of genotoxic DNA breaks, activating the replication checkpoint and formation of DNA repair foci. Thus, our findings suggest that UBXN-3/FAF1 is a specific mediator of CAD, especially in the context of DNA replication since it is not essential for CDC-48/p97-mediated unfolded protein response pathways in the ER or mitochondria.

The detailed analysis of DNA replication factors indeed revealed a crucial role of UBXN-3/FAF1 in the regulation of chromatin-loaded CDT-1, as well as chromatin association of CDC-45 and the GINS complex. Analysis of DNA fibre assays highlights the severe consequences of UBXN-3/FAF1 depletion on the DNA replication programme at single-origin resolution (Fig. 6; Supplementary Fig. 6). Depletion of UBXN-3/FAF1 markedly perturbs progression of replication forks, accompanied by increased numbers of stalled forks and newly fired dormant origins. Notably, this DNA replication defect can be suppressed by codepletion of CDT-1, which thus appears to be a conserved key substrate of UBXN-3/FAF1. Indeed, CHX-chase experiments show that UBXN-3/FAF1 depletion attenuates CDT-1 turnover resulting in stabilized protein levels. Importantly, our binding studies demonstrate that UBXN-3/FAF1 directly interacts with CDT-1 and other ubiquitylated substrates independent of CDC-48 and the UFD-1/NPL-4 cofactor. In accordance with this idea, UBXN-3/FAF1 is localized within the nucleus and associated with chromatin. Together, these data identify UBXN-3/FAF1 as a CDC-48/p97 adaptor and potent mediator of CAD at eukaryotic replisomes (Fig. 7a).

UBXN-3/FAF1 is a member of the UBX family of CDC-48/p97 cofactors, which were proposed to provide substrate specificity^38^. However, only few UBX proteins were convincingly shown to target CDC-48/p97 to compartment-specific degradation pathways. In yeast, Ubx2 and Ubx3 mediate ER-associated degradation or non-proteolytic pathways, respectively^43^,^44^,^45^,^46^. Ubx4 and Ubx5 as well as the mammalian ortholog UBXN7 are involved in the cellular response to ultraviolet (UV) light-induced DNA damage^42^,^47^. In mammalian cells UBX proteins are implicated in cellular functions as diverse as membrane fusion^48^, endolysosomal sorting^49^, lipid metabolism^50^ or ciliogenesis^51^. In *C. elegans*, the functional relevance of UBX proteins is scarcely defined. UBXN-2 is implicated in mitosis, by regulating centrosomal composition^52^. Whereas UBXN-3 joins with UBXN-1 and UBXN-2 in nematode gametogenesis, downregulation of the latter two UBX proteins does not affect DNA replication^30^. Similar to CDC-48 and its cofactor UFD-1/NPL-4, UBXN-3 is localized inside the nucleus during S phase, which is consistent with a role during DNA synthesis. Moreover, cellular fractionation implicates UBXN-3 as well as CDC-48 to be chromatin-associated (Fig. 4a). Deletion of *ubxn-3* does not affect the integrity of the CDC-48^UFD-1/NPL-4^ complex, excluding indirect effects on DNA synthesis. Conversely, the presence of CDC-48, UFD-1 and NPL-4 appears to be important for the correct nuclear localization of UBXN-3.

This study implicates that UBXN-3/FAF1 determines selection of CDC-48/p97 substrates in the nucleus (Fig. 7a). Specifically, UBXN-3/FAF1 facilitates degradation or disassembly of the licensing factor CDT-1 and the GINS complex during DNA synthesis. Interestingly, two recent publications revealed that CDC-48/p97 is required for the release of the MCM helicase together with CDC-45 and GINS (collectively called CMG complex) from chromatin at termination sites of DNA replication^24^,^25^. Thus, the UBXN-3/FAF1-dependent regulation of CDC-45/GINS might be triggered by MCM-7 ubiquitylation and CMG disassembly. However, the localization of functional GFP fusions of MCM-2, MCM-3 as well as MCM-7 was not affected upon UFD-1 or NPL-4 depletion, in contrast to the CMG components CDC-45 and SLD-5 (Supplementary Figs 7b and 3c,d)^16^,^35^. As CMG release requires active DNA synthesis^24^,^25^, delayed disassembly of CMG complexes might also result from perturbed origin firing patterns. The observation that FAF1 depletion causes inefficient DNA synthesis in several human cell lines, which can be restored upon CDT-1 codepletion strongly argues for defects in DNA replication licensing, which unlikely originate from aberrant fork termination. Attenuated disassembly of CMG complexes at termination sites would be expected to result in increased fork termination events. Conversely, analysis of DNA fibres in FAF1-depleted cells revealed a twofold decrease in fork termination frequency (Supplementary Fig. 6e), thus unlikely reflecting a primary role of FAF1 in resolving terminated replication forks in metazoans. In support of this, depletion of two topoisomerase-II orthologs *top-2* and *cin-4* does not result in elevated CDT-1 levels on mitotic chromatin (Supplementary Fig. 7a), thus excluding secondary effects of aberrant replication termination^53^. Importantly, fork stalling and firing of dormant origins are consequences of ATR/CHK-1 checkpoint activation^54^, illustrating occurrence of severe DNA replication stress in FAF1-depleted cells. Although control of CDT-1 activity is the primary mechanism of DNA replication regulation by UBXN-3/FAF1, adjustment of other replication factors might contribute to avoid replication stress. Besides CDT-1, our data show that UBXN-3/FAF1 binds additional ubiquitin conjugates (Fig. 5b,c). Generally, UBXN-3/FAF1 appears to be associated with the chromatin (Fig. 4a). Therefore, UBXN-3/FAF1 presumably acts on several substrates during DNA replication, causing pleiotropic defects on fork progression when absent. This study and our previous work show that codepletion of either CDT-1 or CDC-45/GINS together with UFD-1/NPL-4 is able to suppress checkpoint activation^16^ and fork progression defects, supporting the idea that different CDC-48/p97 substrates are targeted throughout the DNA replication programme. Thus, we propose that UBXN-3/FAF1 cooperates with CDC-48/p97 at distinct time points during DNA replication.

Recently, mutations in the CDC-48/p97-associated protease SPRTN (also called DVC1 or C1orf124) were shown to be directly linked to accelerated aging and cancer susceptibility^22^,^23^. Likewise, aberrations in FAF1 gene expression were identified in cancer patients, however, the causative mechanisms remained unclear^55^,^56^,^57^,^58^,^59^ (and references in Menges *et al.*^26^). This study identifies an unexpected role of UBXN-3/FAF1 in faithful DNA duplication and genome integrity. Since chronic replication stress is known to generate chromosomal alterations associated with cancer as well as reduced proliferative capacity of stem cells^5^,^60^,^61^,^62^, FAF1 misregulation might be linked to oncogenesis and accelerated aging through genome instability. Besides DNA replication, its role in apoptosis and NF-κB signalling further emphasizes that FAF1 represents a potent target for chemotherapy and human genetic studies^26^.

## Methods

### Worm strains

*C. elegans* nematodes were treated according to standard protocols at 20 °C, unless otherwise stated. The Bristol strain N2 was used as wt. Mutants and transgenic animals used in this study are listed in the following:

*cdc-48.1(tm544)II*, *ubxn-3(tm6658)II*, *ubxn-3(tm6658)II*; *hhIs149(unc-119(+)*; *Ppie-1::mCherry::ubxn-3::pie-1-3*′*UTR)*, *unc-119(ed3)III*; *hhIs149(unc-119(+)*; *Ppie-1::mCherry::ubxn-3::pie-1-3*′*UTR)*, *unc-119(ed3)III*; *hhIs177(unc-119(+)*, *Ppie-1::LAP::mcm-7::pie-1-3*′*UTR)*, *unc-119(ed3)III*; *gtIs65(unc-119(+)*, *Ppie-1::LAP::cdc-45::pie-1-3*′*UTR)*, *cdc-48.1(tm544)II*; *gtIs65*, *unc-119(ed3)III*; *gtIs67(unc-119(+)*, *Ppie-1::LAP::sld-5::pie-1-3*′*UTR)*, *cdc-48.1(tm544)II*; *gtIs67*, *unc-119(ed3)III*; *gtIs64(unc-119(+))*, *gtIs64*, *zcIs4(hsp-4::GFP)V*, *cdc-48.1(tm544)II*; *zcIs4(hsp-4::GFP)V*, *zcIs13(hsp-6::GFP)V*, *cdc-48.1(tm544)II*; *zcIs13(hsp-6::GFP)V.*

The transgenic lines *hhIs149* and *hhIs177* were generated for this study. Briefly, *ubxn-3* cDNA was cloned into the pAZCherry(N)^63^, whereas *mcm-7* cDNA was cloned into the pIC26 expression vectors, respectively^64^. The transgenes were delivered and integrated into the *C. elegans* genome by particle bombardment^65^.

### RNAi

RNAi-mediated depletion was achieved using the feeding method^66^. RNAi bacteria were fed either to L1 larvae or starting at the L3/L4 larval stage at 15 °C, until adulthood was reached. dsRNA production was induced with 2 mM Isopropyl-β-D-thiogalactopyranoside (IPTG) (unless otherwise stated) added to the growth media. RNAi constructs were either obtained from the Ahringer or ORFeome WS112 libraries (Geneservice Ltd, available via Source BioScience, UK, catalogue nos. 3318 and 3320); for depletion of *pcn-1* the respective cDNAs was subcloned into the pPD129.36 feeding vector. The empty feeding vector was used as control. Depletion of NPL-4 protein levels was achieved by induction of dsRNA corresponding to the *npl-4.2a* cDNA.

### HU sensitivity

HU sensitivity was monitored by growing worms in the presence of different HU concentrations. Worms were transferred to HU-containing medium after reaching the L4 larval stage to avoid developmental effects. Following RNAi and HU treatment until adulthood, five to six adult worms were kept for 5–6 h on nematode growth medium (NGM) plates for egg laying. The following day, hatched progeny and dead embryos were counted to calculate growth defects. Because of a developmental delay, the analysis of *ubxn-3(tm6658)* mutants was shifted by one day, respectively.

### Lifespan analysis

For lifespan assays, worms were synchronized by bleaching in sodium hypochlorite. Eggs were distributed on RNAi plates. For HU treatment, L4 larvae were transferred to HU-containing RNAi plates. In lifespan assays, first day of adulthood was defined as day 1. Worms were grown at 20 °C and monitored daily for survival by touch-provoked movement and pharyngeal pumping, until death. Animals that crawled off the plates or died owing to internal hatching of progeny or extruded organs were censored.

### Microscopy and image acquisition

For time-lapse microscopy, embryos were extruded from gravid hermaphrodites with the help of injection needles, transferred onto 3% agar pads in M9 buffer before microscopic analysis. An AxioImager.M1/Z1 microscope equipped with an AxioCam MRm camera (Carl Zeiss) was used for image acquisition. Time-lapse recordings in 90-s intervals were acquired using 2 × 2 mono binning to avoid photo bleaching and toxicity. To allow direct comparison of signal intensities, images were recorded under identical conditions. Analysis of time-lapse recordings was done in the AxioVision 4.7 software. Timing of cell division was estimated as follows: for the estimation of cell division timing distinct cell-cycle stages (nuclear envelope breakdown (NEBD), completion of mitotic furrow ingression (cytokinesis)) where determined morphologically from differential interference contrast (DIC) images. Time intervals in between theses cell-cycle stages were used to calculate the duration of mitosis (time elapsed between NEBD and cytokinesis of one-cell division) and duration of S phase (time elapsed between cytokinesis and NEBD of two subsequent divisions). The P1 delay refers to a combined estimation of the delay in cell-cycle progression of the P1 cell relative to the AB cell. The P1 delay represents the average delay calculated from the time difference between NEBD on one hand and the completion of cytokinesis in the AB and P1 cell on the other hand^27^,^28^. Processing of selected pictures was done in Adobe Photoshop CS4. Images of immunostainings were also acquired with AxioImager.M1/Z1 and AxioCam MRm but using full resolution of the camera. Confocal images were acquired using the Yokogawa CSU-X1 spinning disc module mounted to a Nikon Ti-E microscope stand, operated by Volocity software (PerkinElmer). Z-stacks were recorded with 200 nm distances between optical sections and projected into one single image. Microscopy of DNA fibres was done using a Leica DMRB microscope with a DFC360FX camera.

### Antibody production

cDNAs encoding *cdt-1*, *npl-4.2* and *ubxn-3b* were cloned into the pET-21a expression vector (Addgene). Recombinant proteins were expressed in *Escherichia coli* strain BL21cp (Stratagene) and purified using the ÄKTApurifier system (GE Healthcare) or bench-top method using Ni-NTA agarose (Qiagen). His-tagged purified proteins were used for immunization of rabbits and anti-sera were affinity purified using respective Strep-II-tagged recombinant proteins (BioGenes).

### Immunotechniques

Immunostaining of early embryos was done essentially according to the ‘freeze-crack' protocol. Gravid worms were dissected onto polylysine-coated slides (Thermo Scientific) and frozen in liquid nitrogen, followed by incubation in methanol at −20 °C for 20 min and in acetone at −20 °C for 20 min. After rehydration in PBS and blocking in 5% BSA, embryos were incubated with primary antibody overnight at 4 °C (anti-UBXN-3 1:8,000, anti-CDT-1 1:300, anti-RAD-51 1:400 (Novus), anti-FK2-ubiquitin 1:500 (Millipore) and anti-α-tubulin 1:200 (Sigma-Aldrich, clone DM1A)). Incubation with the fluorescently labelled secondary antibodies (Life Technologies) was done at room temperature for 1 h (1:400). Embryos were then mounted in DAPI Fluoromount-G medium (SouthernBiotech). For western blotting, purified proteins and worm lysates were separated by SDS–polyacrylamide gel electrophoresis (SDS–PAGE) and transferred to nitrocellulose membranes (Whatman, Protran). Membranes were blocked in 3% milk solution and incubated with the primary antibodies overnight at 4 °C in Roti-Block (Roth; anti-CDT-1 1:5,000, anti-UFD-1 1:50,000, anti-CDC-48 1:50,000, anti-NPL-4 1:10,000, anti-UBXN-3 1:50,000, anti-GFP 1:5,000 (Clonetech), anti-tubulin 1:5,000 (Sigma-Aldrich, clone DM1A), anti-FK2-ubiquitin 1:2,000 (Millipore), anti-histone3 1:5,000 (Abcam) and anti-mCherry 1:2,500 (Abcam)). Incubation with fluorescently labelled secondary antibodies (1:10,000) was done at room temperature, before detection of signals using the Li-Cor Odyssey scanner. Quantification of signal intensities was done using the Odyssey V3.0 software (Li-Cor). The uncropped versions of western blots that have been used to assemble the main figures are collected in Supplementary Fig. 8.

### Preparation of worm lysates

Synchronized L1 larvae were kept on control or RNAi plates at 15 °C until they reached adulthood. Embryos of gravid worms were collected by bleaching and re-suspended in 2 × Laemmli buffer. Embryos were sonicated twice for 15 s (Bandelin, Microtip MS 1.5), incubated at 95 °C for 5 min, and centrifuged at 14,000*g* for 5 min before SDS–PAGE analysis. Whole-worm lysates used for subsequent co-IP were prepared as follows: synchronized L1 larvae were grown on NGM-agar plates with OP50 bacteria until they reached adulthood. Worms were collected and washed once in lysis buffer (50 mM Tris (pH 7.2), 50 mM NaCl, 1 mM EDTA and protease inhibitors (Roche)) before shock-freezing in liquid nitrogen. Subsequently, thawed samples were subjected to sonication (four times for 30 s, on ice; 50% power; Sonopuls UW 2200, Bandelin). Samples were centrifuged at 14,000*g* for 5 min and supernatant subsequently incubated with magnetic Dynabeads Protein A (Novex Life Technologies). Protein concentrations were calculated by extinction at 280 nm using the NanoDrop 8000 (Thermo Scientific).

### Cellular fractionation of embryos

Purification of nuclei from embryos was achieved according to published protocols with some modifications^67^,^68^. Briefly, freshly collected embryos were disrupted in a Wheaton stainless steel homogenizer (7 ml capacity) in nuclear preparation buffer (NPB; 20 mM HEPES, 20 mM KCl, 3 mM MgCl_2_, 2 mM EDTA, 0.5 M sucrose, 1 mM dithiothreitol, 1 mM phenylmethyl sulphonyl fluoride and protease inhibitors (Roche)). Efficient lysis was monitored under the microscope. Cellular debris was collected by three subsequent centrifugation steps at 100*g* for 1 min at 4 °C. Nuclei were collected by centrifugation at 4,000*g* for 5 min at 4 °C. The supernatant contains represents the cytosolic fraction. The nuclei in the pellet were washed twice in detergent buffer (DTB; same as NPB plus 0.25% Igepal, 0.1% Triton X-100) and collected as before at 4,000*g* centrifugation. After resuspension in one-fourth volume nuclear extraction buffer (NEB; 20 mM HEPES, 350 mM NaCl, 5 mM CaCl_2_, 2 mM EDTA, 25% glycerol, 1 mM dithiothreitol, 1 mM phenylmethyl sulphonyl fluoride and protease inhibitors (Roche)) of the cytosolic fraction, the nuclear fraction was split in two halves. One half was kept on ice while the second half was subjected to sonication (twice for 20 s at 90% power, Bandelin Microtip). Nucleoplasm and chromatin fraction were separated by centrifugation at 20,000*g* for 10 min at 4 °C. Supernatant contains soluble proteins, pellet contains chromatin-associated proteins^69^. After resuspension of the chromatin pellets in NEB all samples were diluted in Laemmli buffer and subjected to sonication (twice for 20 s at 90% power, Bandelin Microtip) before SDS–PAGE analysis.

### Protein interaction studies

For co-IP analysis, magnetic Dynabeads Protein A (Novex Life Technologies) were used following the manufacturer's protocol. Dynabeads were mixed with the antibody of choice (10 μg), diluted in 200 μl of PBST (0.01% Tween 20), and crosslinked using BS3 (Thermo Scientific). After crosslinking, worm lysate (1 mg total protein) was added and incubated with the Dynabeads for 30 min at room temperature. After three times washing with washing buffer (50 mM Tris (pH 7.2), 1 mM EDTA, 150 mM NaCl, 10% glycerol and protease inhibitors (Roche)), bound proteins were eluted in Laemmli buffer without β-mercaptoethanol and heated for 10 min at 70 °C. β-Mercaptoethanol was added to the eluate just before SDS–PAGE analysis. For analysis of UBXN-3 functional domains, His-tagged proteins were expressed and purified from *E. coli* using Ni-NTA agarose (Qiagen) and PD10 gel-filtration columns (GE Healthcare). Isolated proteins were snap-frozen in liquid nitrogen and stored at −80 °C. Synchronized adult worms were lysed by sonication (Bandelin Sonopuls) in lysis buffer (50 mM Tris (pH 7), 150 mM NaCl, 0.5 mM EDTA, 5 mM MgCl2, 0.1% Igepal, 15 mM imidazole, protease inhibitors (Roche) and 5 mM iodoacetamide). Proteins coupled to Ni-NTA (50 μl slurry per 1 mg protein) or Ni-NTA agarose alone were incubated with worm lysate (20 × excess total protein over His-tagged peptides) for 30 min at room temperature. After three times washing with washing buffer (same as lysis buffer with 250 mM NaCl), bound proteins were eluted from agarose beads with elution buffer (50 mM Tris (pH 7), 8 M urea, 100 mM NaH_2_PO_4_ and 0.1% Igepal). Remaining proteins were eluted at 95 °C in Laemmli buffer.

Co-IP analysis in human cells was achieved via anti-HA agarose beads following the manufacture's protocol. Twenty-four hours before collecting HEK293 cells, expression of the p97-E578Q mutant^42^ was induced by doxycycline (1 μg ml^−1^ final). In addition, cells were transiently transfected with a plasmid driving the expression of HA-tagged FAF1 or HA as control (pIRESpuro2b vector). HA beads were washed twice in IP buffer (50 mM Tris (pH 8.0), 150 mM NaCl and 0.05% NP-40) and incubated with nuclear lysates for 3 h at 4 °C. Nuclear lysates were prepared^69^ and after five times washing with IP buffer (supplemented with protease inhibitor cocktail (Roche)) bound proteins were eluted in 2 × Laemmli buffer (with β-mercaptoethanol) and boiled for 10 min at 95 °C.

### Cell line cultivation, transfection and analysis

Human osteosarcoma (U2OS) cells, human embryonic kidney 293 (HEK293) cells and human bladder cancer (T24) cells (cell lines were purchased at ATCC, UK (CRL-1573, HTB-96 and HTB-4]) were cultured in six-well plates, 60- or 100-mm dishes in DMEM supplemented with 10% fetal bovine serum and 1% penicillin–streptomycin in a 5% CO_2_ atmosphere at 37 °C. Cells were transfected with the indicated siRNAs (final concentration of 20 or 50 nM) using Lipofectamine RNAiMAX (Invitrogen) transfection reagent according to manufacturer's instructions at 50–60% cell confluency.

For preparation of lysates, cells were cultured in 60 or 100 mm dishes and collected 72 h after transfection. Cells were trypsinized, washed with PBS and the cell pellets were re-suspended in lysis buffer (50 mM Tris-HCl (pH 7.5), 120 mM NaCl, 20 mM sodium fluoride, 1 mM EDTA, 6 mM EGTA, 1% Nonidet P-40 and 10% glycerol), and supplemented with protease inhibitor (Roche, complete EDTA free), phosphatase inhibitor (Roche, PhosStop) and *N*-ethylmaleimide (20 mM). The protein content was estimated using Bio-Rad protein assay reagents. Lysates were resolved in polyacrylamide gels using standard protocols. Membranes were blocked with 5% non-fat dry milk for 1 h with agitation at room temperature. The proteins were detected with respective primary and secondary antibodies and visualized on X-ray film.

To analyse protein CDT-1 turnover, HEK293 cells were grown in DMEM media before addition of CHX (50 μg ml^−1^ final concentration). At the selected time points, the cells were collected and cell lysates were prepared as described above, followed by western blot analysis.

### DNA fibre assay

Asynchronous U2OS, HEK293 or T24 cells were pulse labelled with 30 μM of CldU (Sigma-Aldrich) for 30 min, washed three times with warm PBS and then 250 μM of IdU (Sigma-Aldrich) pulse were given for an additional 30 min. Labelling of replication forks was terminated by treating the cells with ice-cold PBS. Cells were lysed, and DNA fibres were spread onto glass slides, fixed with methanol and acetic acid, denatured with HCl, blocked with 2% BSA and stained with anti-rat and anti-mouse 5-bromo-2′-deoxyuridine (BrdU) that specifically recognize either CldU or IdU. Anti-rat Cy3 (dilution 1:300, Jackson Immuno Research) and anti-mouse Alexa 488 (dilution 1:300, Molecular Probes) were used as the respective secondary antibodies. The analysis of DNA tract length is exclusively based on measurement of symmetric tracts, displaying no statistical differences in the lengths of respective CldU- and IdU-single tracts^22^,^70^.

### siRNA sequences

siFAF1 #3 (5′-CUGUACUGUUGGAGAGAUUAATT-3′, Microsynth); siFAF1 #5 (5′-AAGGACGAGGAUGAACGUGAATT-3′, Microsynth); siFAF1 #6 (5′-AACAUUGACGAAGCUAUUACATT-3′, Microsynth); siFAF1 #7 (5′-ACCAACGUGUUCUGCUCACAATT-3′, Microsynth); siFAF1 3′UTR #2 (5′-ACACGCUCGUCUCACUCA-3′, Microsynth); siCdt1 (5′-AACGUGGAUGAAGUACCCGACTT-3′, Microsynth); and siNS (negative siRNA control, Qiagen).

### Antibodies against human proteins

Rabbit anti-CDT-1 (Bethyl A300-786A, 1:1,000), rabbit anti-FAF1 (Cell Signaling #4932S, 1:1,000), mouse anti-β-actin (Abcam Ab6276, 1:10,000), mouse anti-p97 (Abcam Ab11433, 1:1,000), mouse anti-ATM (Sigma-Aldrich A1106, 1:2,000), rabbit anti-phospho ATM ser1941 (Abcam Ab81212, 1:5,000), rabbit anti-ATR (Bethyl A300-A137A, 1:1,000), rabbit anti-phospho ATR ser428 (Millipore ABE389, 1:1,000), mouse anti-CHK-1 (Cell Signaling 2360, 1:1,000), rabbit anti-phospho CHK-1 ser345 (Cell Signaling 2348, 1:1,000), rabbit anti-CHK2 (Cell Signaling 2662, 1:1,000), rabbit anti-phospho CHK2 Thr68 (Cell Signaling 2661), rabbit anti-phospho H2AX Ser139 (Novus Biologicals NB100-2280, 1:1,000), rat anti-HA (Roche, clone 3F10, 1:5,000) and mouse anti-PCNA (Abcam Ab29, 1:1,000). Anti-rabbit and anti-mouse horse radish peroxidase-conjugated secondary antibodies were purchased from Sigma-Aldrich (A9169 and A9044, respectively, 1:10,000). Membranes were activated using Thermo Scientific SuperSignal West Pico and Dura substrates and developed accordingly using standard protocol.

### Statistical analysis

Statistical analysis was performed using Excel (Microsoft), Prism (GraphPad) and ImageJ software. Statistical significance was calculated with two-tailed paired Student's *t*-test. Statistical significance of *C. elegans* lifespan was estimated using the log-rank Mantel–Cox test. Asterisks indicate statistical significances according to common conventions.

## Additional information

**How to cite this article:** Franz, A. *et al.* Chromatin-associated degradation is defined by UBXN-3/FAF1 to safeguard DNA replication fork progression. *Nat. Commun.* 7:10612 doi: 10.1038/ncomms10612 (2016).

## Supplementary Material

Supplementary Information Supplementary Figures 1-8 and Supplementary Table 1
