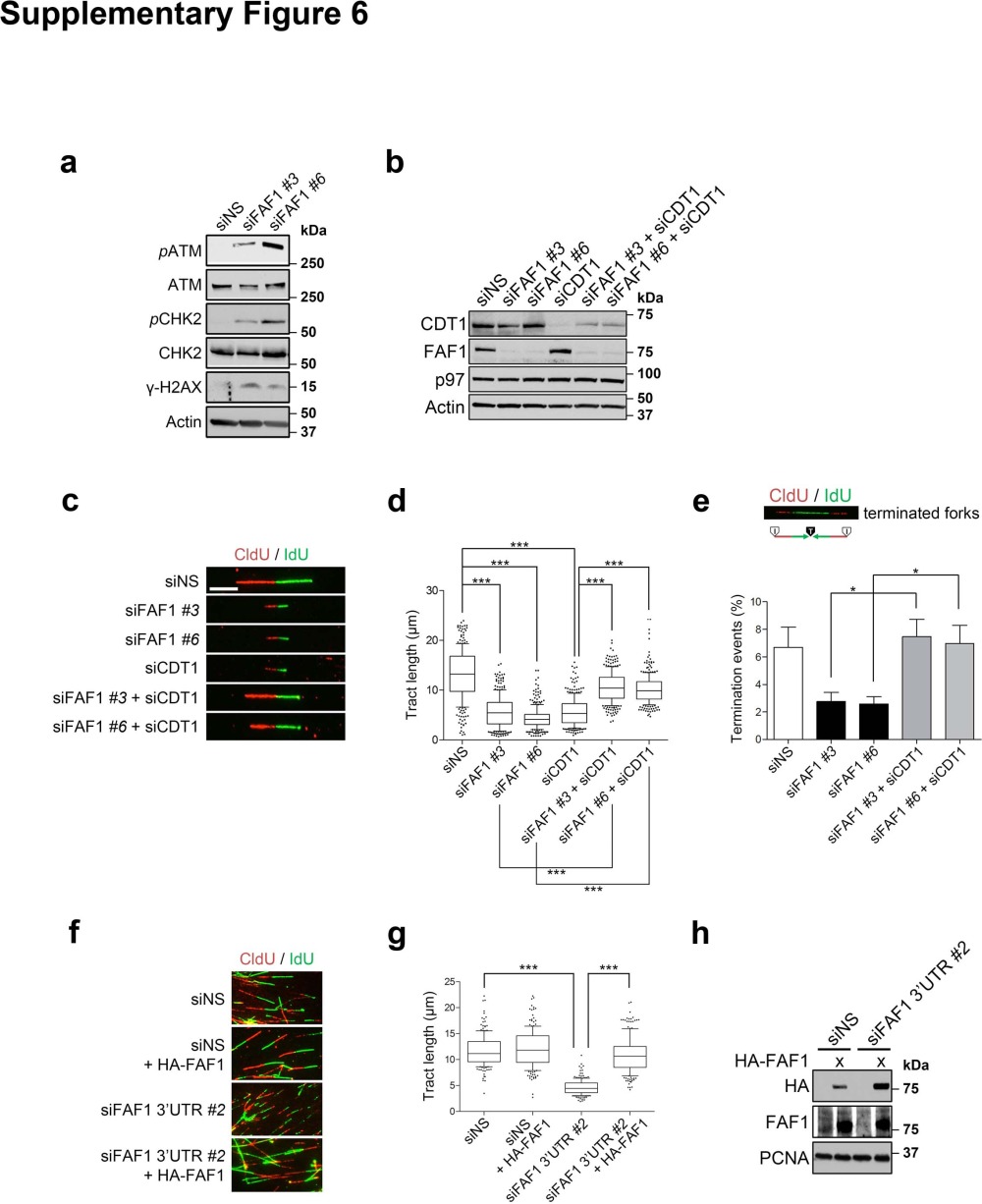# Erratum: Chromatin-associated degradation is defined by UBXN-3/FAF1 to safeguard DNA replication fork progression

**DOI:** 10.1038/ncomms11593

**Published:** 2016-05-17

**Authors:** André Franz, Paul A. Pirson, Domenic Pilger, Swagata Halder, Divya Achuthankutty, Hamid Kashkar, Kristijan Ramadan, Thorsten Hoppe

Nature Communications 7: Article number: 1061210.1038/ncomms10612 (2016); Published: 02042016; Updated: 05172016.

This Article contains errors in the labelling of Fig. 5 and Supplementary Fig. 6, the former of which was introduced during the production process. In Fig. 5b, an ‘x' indicating the presence of UBXN-3(SGG)-HIS in lane 6 was inadvertently omitted. In Supplementary Fig. 6g, the fourth bar from the left should have been labelled as ‘siFAF1 3′UTR #2+HA-FAF1'. The correct version of both figures appear below.

Figure 5:


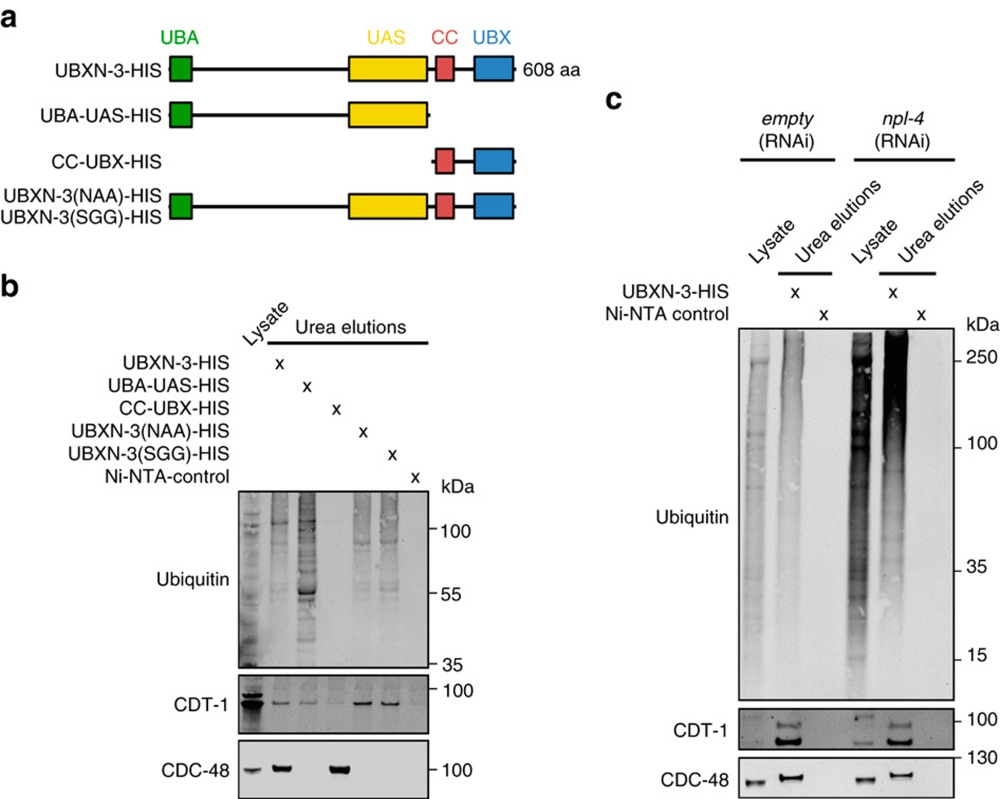


Supplementary Figure 6: