# Supplementary material for: Chromatin-associated degradation is defined by UBXN-3/FAF1 to safeguard DNA replication fork progression
Source: Nat Commun. 2016 Feb 4;7:10612. doi: 10.1038/ncomms10612 (PMC4743000; doi:10.1038/ncomms10612)
Supplement: Supplementary Information — Supplementary Figures 1-8 and Supplementary Table 1 [file ncomms10612-s1.pdf]

Supplementary Figure 1

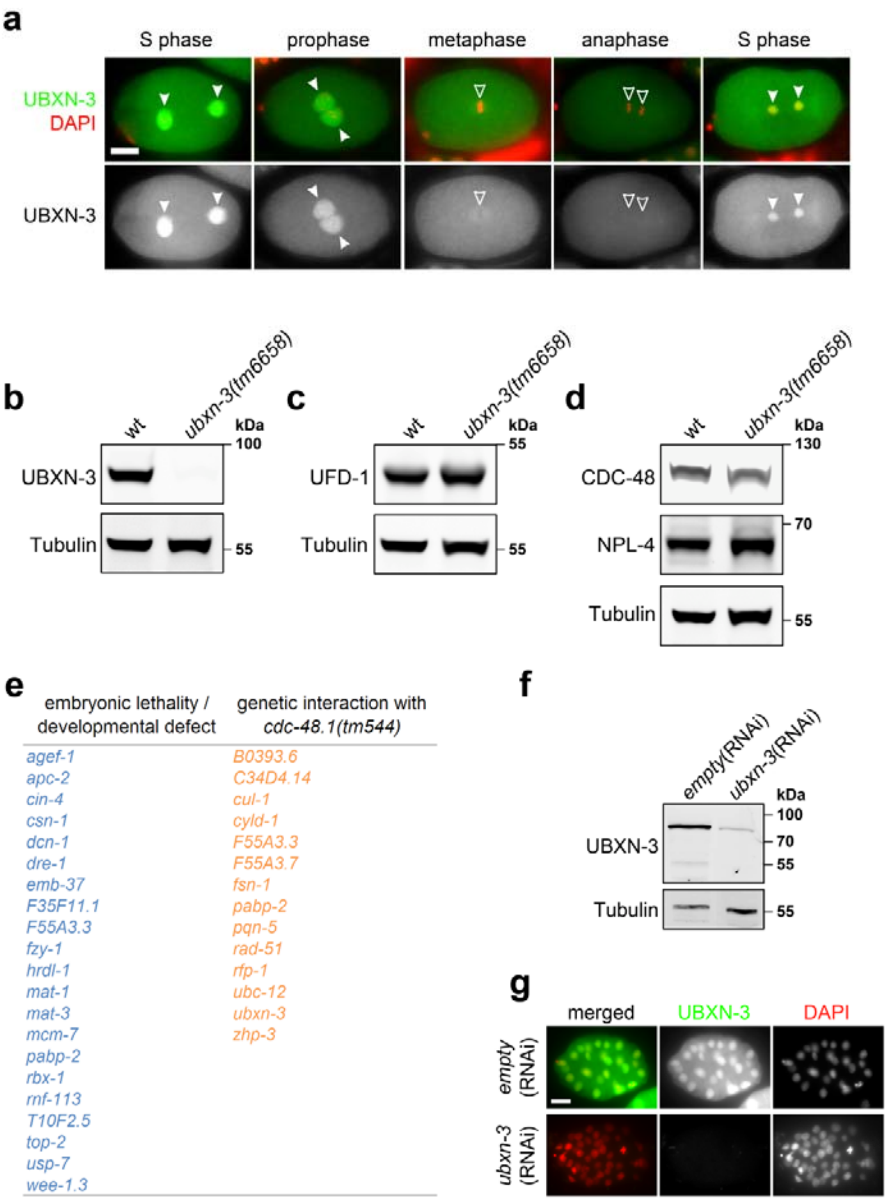

Supplementary Figure 1. Cell cycle-dependent localization of UBXN-3 in *C. elegans* embryos

(a) Immunostainings of *C. elegans* embryos at indicated time points during the first mitotic division. UBXN-3 (green) and DAPI (red) staining is shown as merge images and in separate channels. Scale bar represents 10 µm.

(b, c, d) Western blot analysis of indicated proteins in wt and the *ubxn-3(tm6659)* deletion mutant.

(e) List of genes causing embryonic lethality or developmental defects when depleted in the wt background (blue) or in the *cdc-48.1(tm544)* deletion mutant (orange).

(f) Western blot analysis of whole worm protein extracts of animals treated with *empty* control or *ubxn-3* RNAi.

(g) Immunostaining of embryos depleted for empty control or *ubxn-3* by RNAi. UBXN-3 (green) and DAPI (red) staining is shown as merge images and in separate channels. Scale bar represents 10 µm.

Supplementary Figure 2

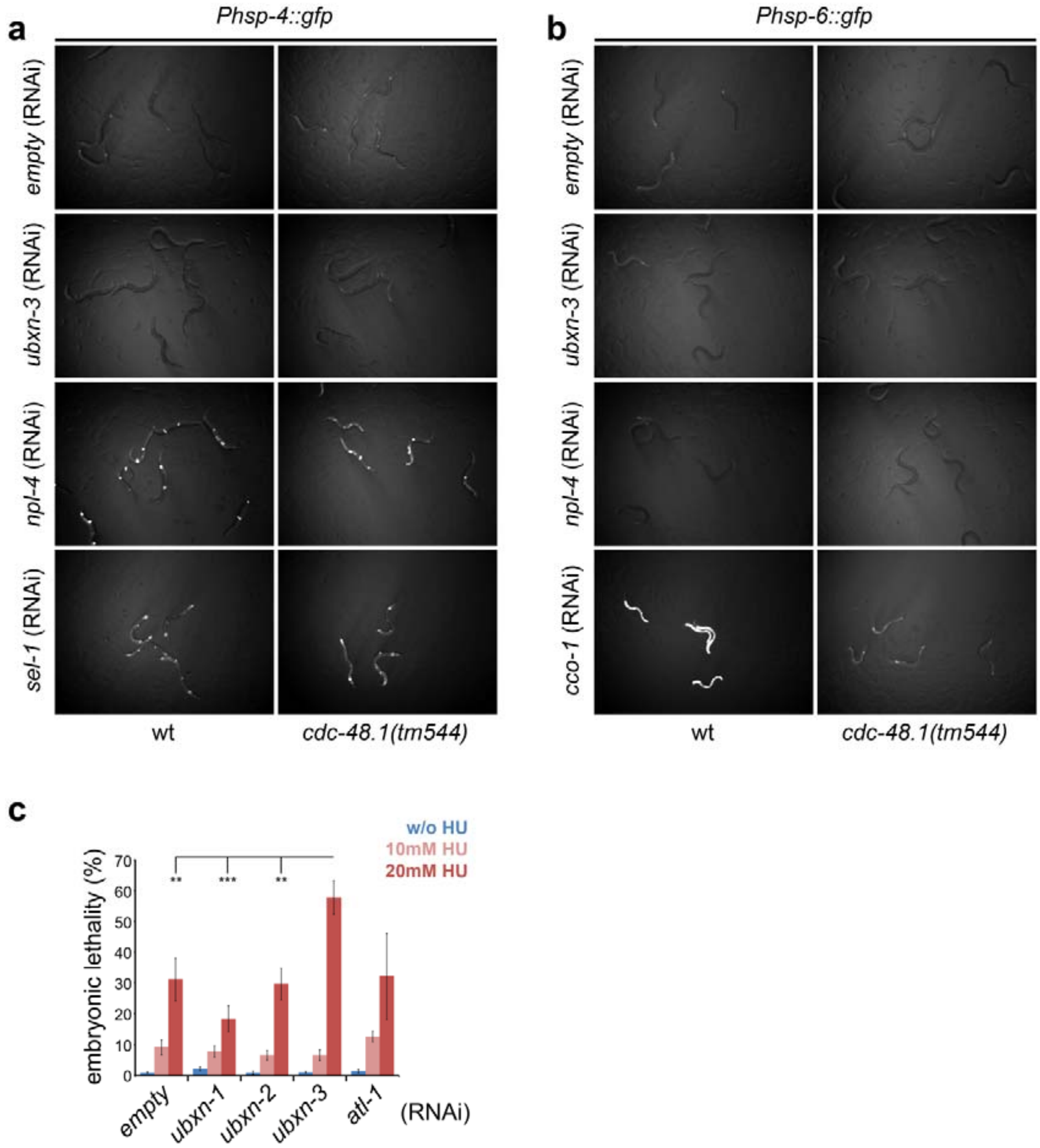

Supplementary Figure 2. Depletion of *ubxn-3* does not result in activation of the UPR In the ER (UPR^{ER}) or mitochondria (UPR^{Mito})

(a, b) Worms expressing the UPR^{ER} reporter *Phsp-4::gfp* or the UPR^{Mito} reporter *Phsp-6::gfp* were depleted for indicated genes by RNAi in the wt or *cdc-48.1(tm544)* mutant background. Representative images of the GFP channel are shown.

(c) HU sensitivity after RNAi depletion of indicated genes in wt worms. Error bars show standard error of the mean (SEM). Mean values were calculated from eight replicates. The double asterisk indicates a P-value of ≤ 0,001, and the triple asterisk indicate P-values of ≤ 0,0001 in Students T-test.

Supplementary Figure 3

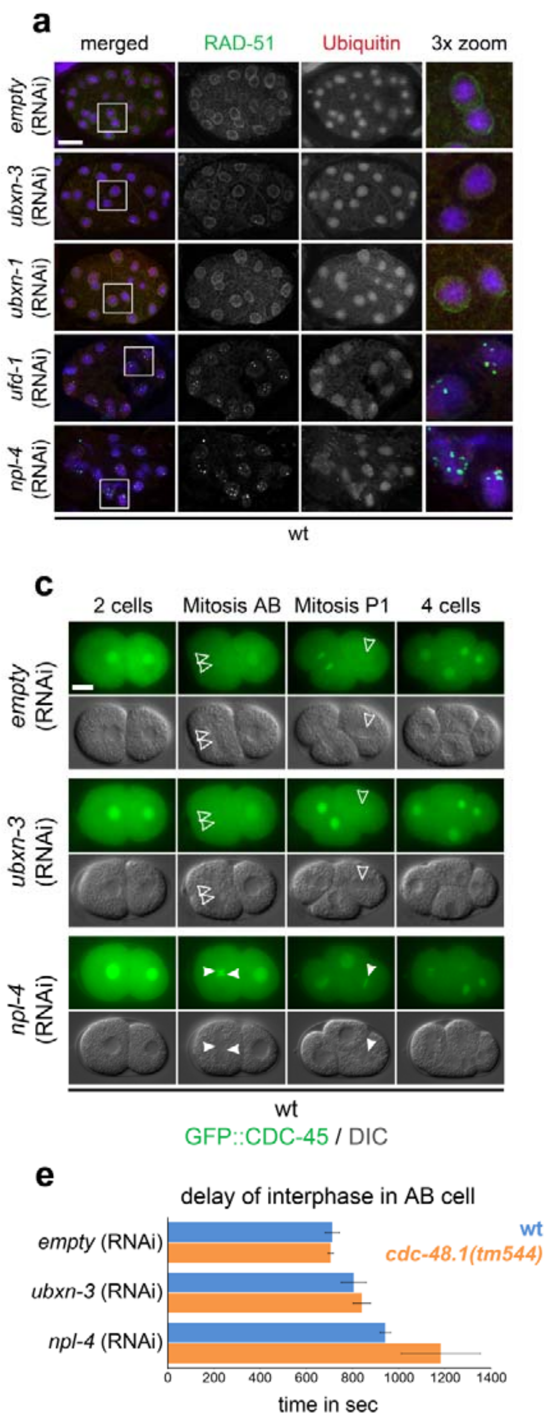

Supplementary Figure 3. Distribution of CDT-1 and CDC-45/GINS in embryos depleted for *ubxn-3*

(a) Representative projections of confocal z-stacks of embryos RNAi depleted for indicated genes in respective genetic backgrounds. Embryos were immunostained for RAD-51 (green) and conjugated ubiquitin (FK2) (red). DNA was stained with DAPI (blue). The boxed area is three times magnified (3x zoom). Scale bars represent 10 μm.

(b) Immunostainings of one-cell *C. elegans* embryos treated with indicated RNAi-feeding constructs. CDT-1 (green), tubulin (red) and DAPI (blue) staining is shown as merge images and in separate channels. The boxed area is magnified by two times (2x zoom). Empty arrowheads indicate wild-type-like CDT-1 levels, filled arrowheads highlight elevated CDT-1 levels on anaphase chromatin. Scale bar represents 10 μm.

(c, d) Representative images of time-lapse recordings of wt embryos expressing GFP::CDC-45 (green, left panel) or GFP::SLD-5 (green, right panel) depleted for *empty* control, *ubxn-3*, and *npl-4*. Each image series shows selected cell-cycle phases of the mitotic division in the AB and P1 cell, respectively. Empty arrowheads point on wild-type-like CDC-45 and SLD-5 localization, filled arrowheads indicate persisting CDC-45 and SLD-5 protein on mitotic chromatin. DIC is shown in grey. Scale bars represent 10 μm.

(e) Quantification of duration of interphase in the AB cell wt (blue) and *cdc-48.1(tm544)* (orange) embryos depleted for *empty*, *ubxn-3*, and *npl-4* by RNAi. Time is shown in seconds (sec). Evaluation of cell division timing was done in embryos expressing GFP::CDC-45 or GFP::SLD-5. Mean values are based on four to six individual recordings per condition.

Supplementary Figure 4

a

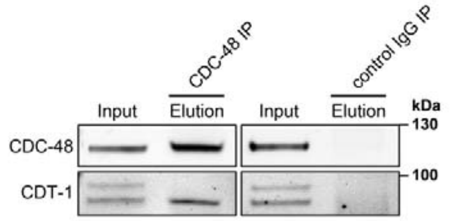

Supplementary Figure 4. CDC-48 interacts with CDT-1 *in vivo*

(a) wt worm lysates were incubated with anti-CDC-48 specific antibodies coupled to protein A dynabeads for immunoprecipitation and subsequently analyzed by western blotting for CDC-48 and CDT-1.

Supplementary Figure 5

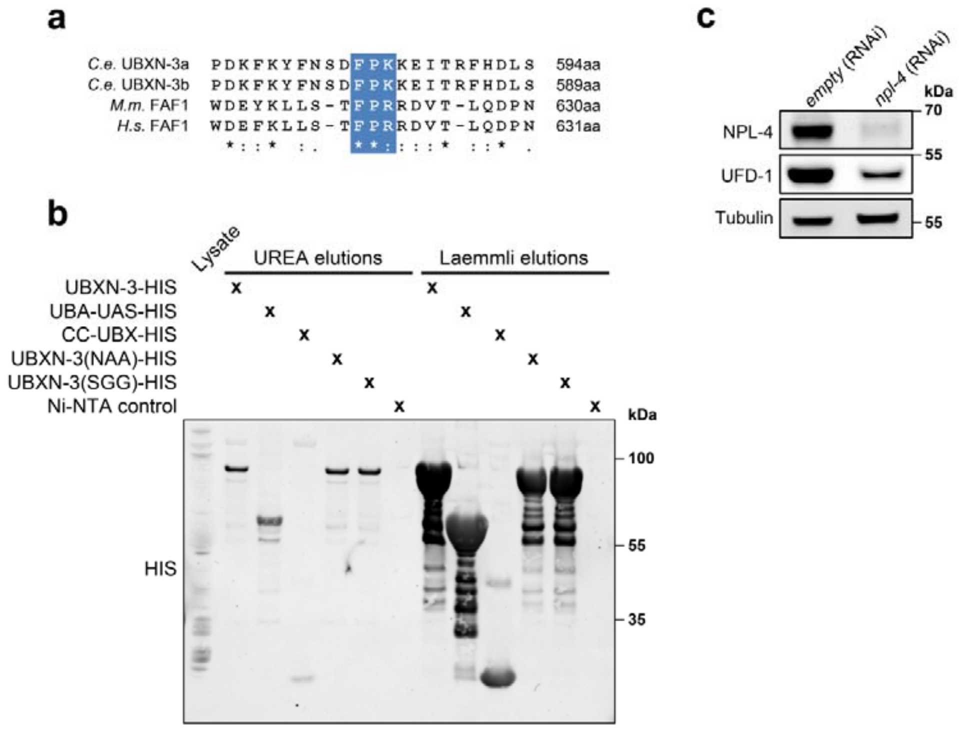

Supplementary Figure 5. Efficiency of RNAi-mediated NPL-4 depletion

(a) Protein sequence alignment of *C. elegans* UBXN-3 and orthologs from *M. musculus* and *H. sapiens*. Conservation of the FPK/FPR motif in the UBX domain is highlighted in blue. The position in the respective amino-acid (aa) sequences is shown on the right.

(b) Worm lysates were incubated with the indicated, recombinant UBXN-3 variants coupled to Ni-NTA beads and analyzed by western blotting for elution of HIS-tagged proteins upon application of UREA or Laemmli buffer respectively.

(c) Western blot analysis of worm lysates depleted for indicated genes by RNAi were immunoblotted for NPL-4, UFD-1, and Tubulin.

Supplementary Figure 6

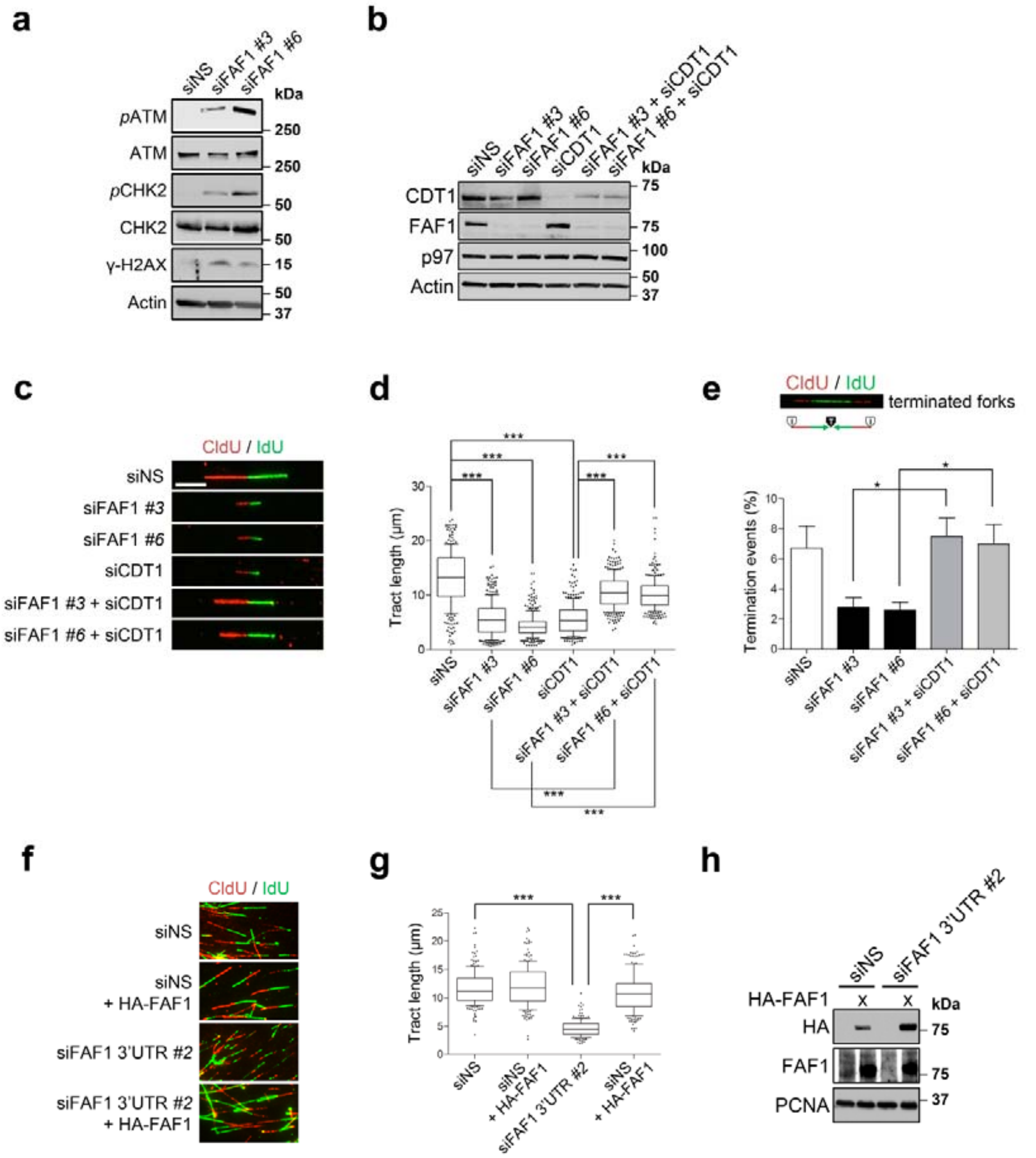

Supplementary Figure 6. FAF1 function in DNA replication is conserved in human cells

(a) Western blot analysis of DNA repair pathway activation by phosho-specific antibodies in HEK293 cells transfected with indicated siRNAs.

(b) Western blot analysis of HEK293 cell lysates transfected with indicated siRNAs. Efficiency of siRNA mediated depletion is shown for FAF1 and CDT1 protein levels.

(c, d) Representative images of microscopic analysis of DNA replication fibers by molecular combing in HEK293 cells after indicated siRNA transfection. CldU (first pulse) incorporation is shown in red, incorporated IdU (second pulse) in green. Quantification of replicated DNA tract-lengths after indicated siRNA transfection in HEK293 cells. Tract-length was determined for 100 forks per condition and experiment. The experiment was repeated in three replicates.

(e) Quantification of fork termination events during 2nd label (IdU) incorporation after indicated siRNA transfection in HEK293 cells. The cartoon illustrates the microscopic manifestation of terminated forks in the DNA fiber assay. I indicates sites of replication initiation, whereas T indicates the site of fork termination. Fork termination was determined for 400 forks per condition and experiment. The experiment was repeated in three replicates.

(f-h) Representative images of microscopic analysis of DNA replication fibers by molecular combing in HEK293 cells after indicated siRNA transfection in combination without/with expression of siRNA-resistant HA-FAF1. Quantification of replicated DNA tract-lengths after indicated siRNA transfection in HEK293 cells. Tract-length was determined for 100 forks per condition and experiment. The experiment was repeated in

three replicates. Western blot analysis confirms the expression of HA-FAF1 and its resistance towards siRNA-mediated depletion.

Whiskers box plots show mean values and data within the 10-90 percentile. Error bars represent standard deviation. The single asterisk indicates a P-value of ≤ 0,05, and the triple asterisk indicate P-values of ≤ 0,0001. Scale bar represents 5 µm.

Supplementary Figure 7

a

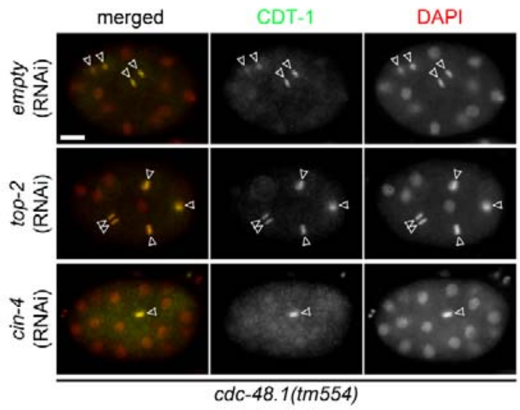

b

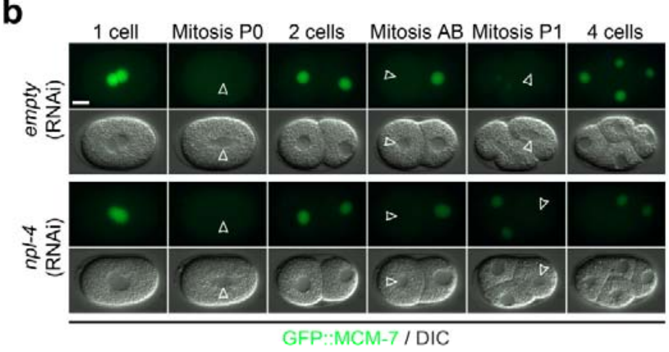

Supplementary Figure 7. GFP::MCM-7 localization is unaffected upon *npl-4(RNAi)*

(a) Immunostainings of *cdc-48.1(tm544) C. elegans* embryos treated with indicated RNAi-feeding constructs. CDT-1 (green) and DAPI (red) staining is shown as merge images and in separate channels. Empty arrowheads indicate wild-type-like CDT-1 levels.

(b) Representative images of time-lapse recordings of wt embryos expressing GFP::MCM-7 (green) depleted for *empty* control and *npl-4*. Each image series shows selected cell-cycle phases of the mitotic divisions between the one and 4 cell stages, respectively. Empty arrowheads point on wild-type-like MCM-7 localization. DIC is shown in grey.

Scale bars represent 10 µm.

Supplementary Figure 8

uncropped blots of Figure 1

Fig. 1b CDC-48 blot

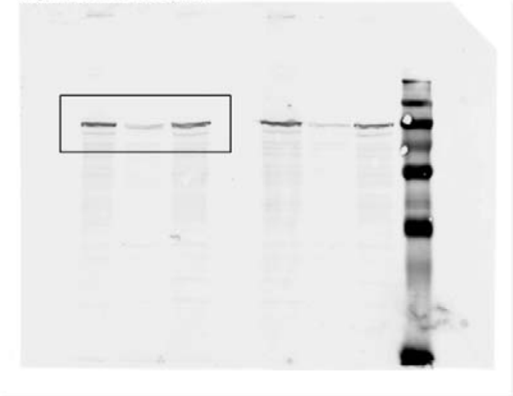

Fig. 1b Tubulin blot

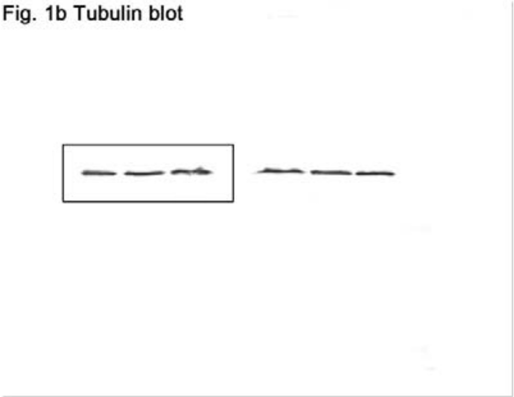

uncropped blots of Figure 2

Fig. 2c mCherry blot

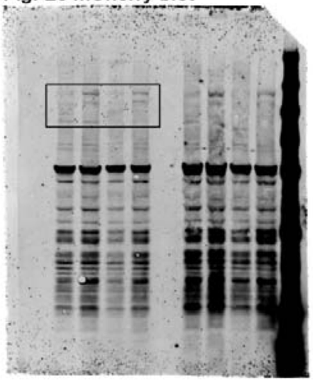

Fig. 2c UBXN-3 blot

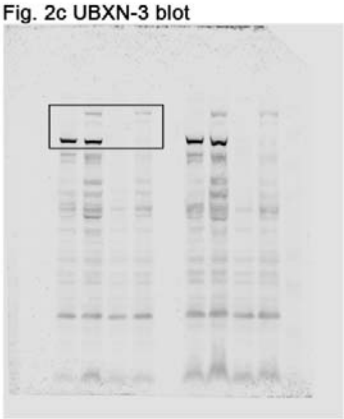

Fig. 2c Tubulin blot

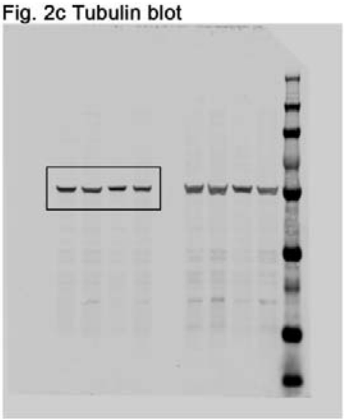

uncropped blots of Figure 4

Fig. 4a CDC-48 blot

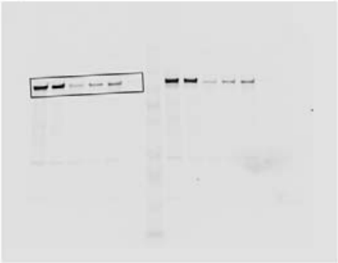

Fig. 4a UBXN-3 blot

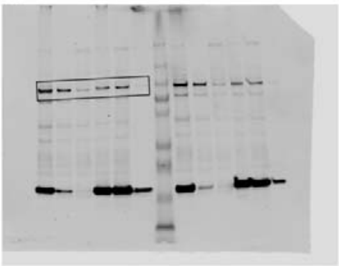

Fig. 4a CDT-1 blot

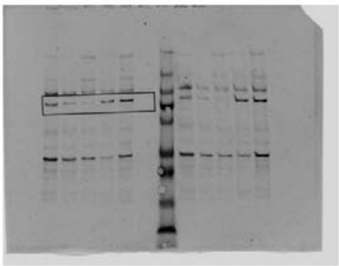

Fig. 4a Tubulin blot

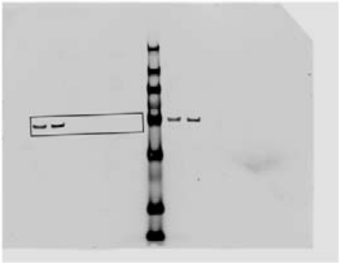

Fig. 4a Histone 3 blot

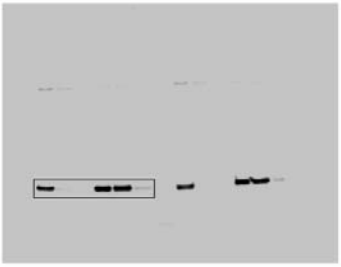

Fig. 4b CDC-48 and UBXN-3 blots

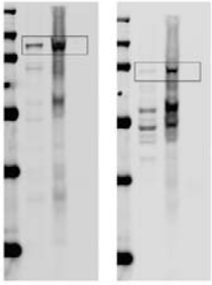

Fig. 4c UBXN-3 and CDT-1 blots

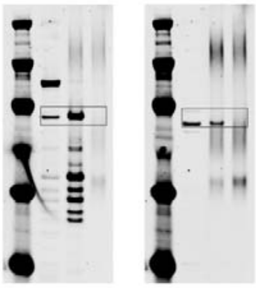

Fig. 4d CDC-48/CDT-1 and UBXN-3 blots

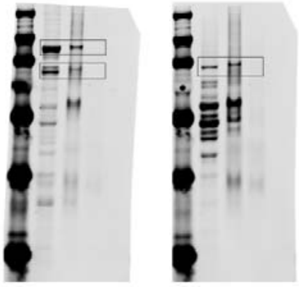

Fig. 4e UBXN-3 blot

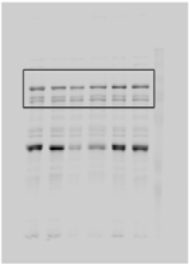

Fig. 4e UFD-1 blot

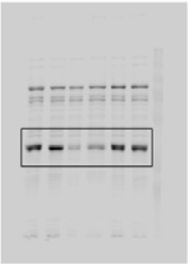

Fig. 4e NPL-4 blot

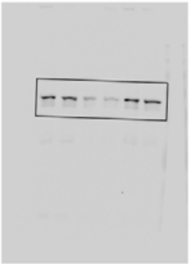

Fig. 4e Tubulin blot

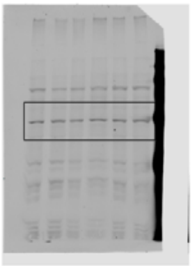

uncropped blots of Figure 5

Fig. 5b Ubiquitin blot

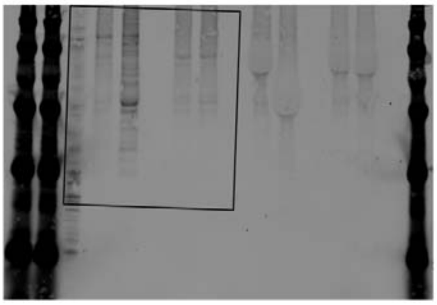

Fig. 5b CDT-1 blot

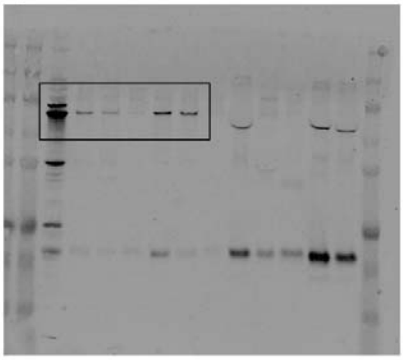

Fig. 5b CDC-48 blot

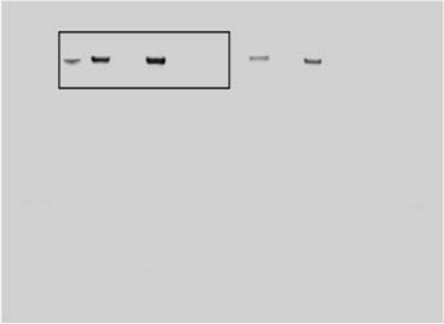

Fig. 5c Ubiquitin blot

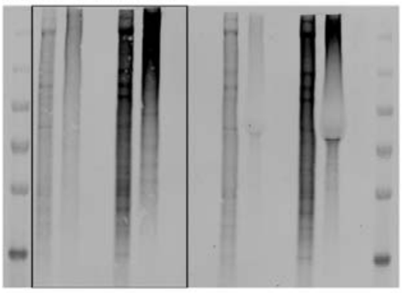

Fig. 5c CDT-1 blot

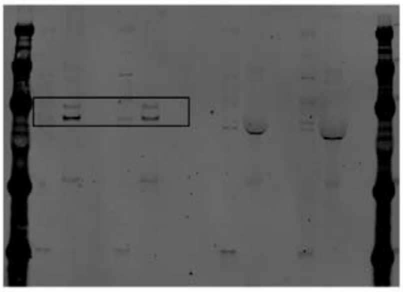

Fig. 5c CDC-48 blot

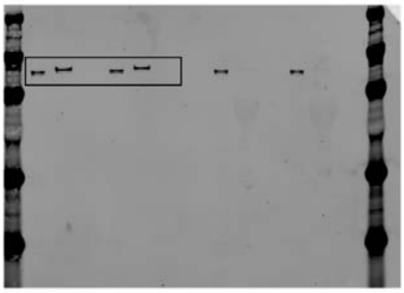

uncropped blots of Figure 6

Fig. 6a FAF1 blot

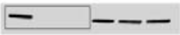

Fig. 6a p97 blot

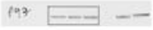

Fig. 6a pATR blot

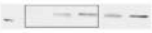

Fig. 6a ATR blot

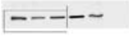

Fig. 6a pCHK1 blot

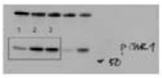

Fig. 6a CHK1 blot

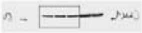

Fig. 6a Actin blot

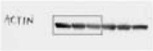

Fig. 6c FAF1 blot

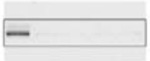

Fig. 6c Actin blot

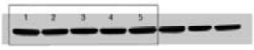

Fig. 6j CDT1 blot

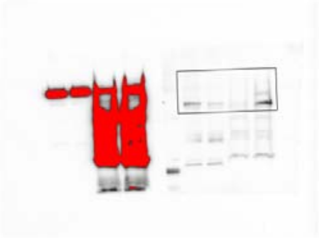

Fig. 6j p97 blot

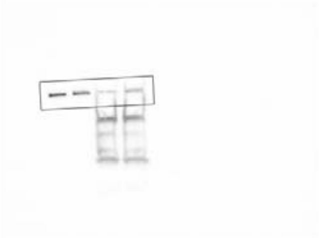

Fig. 6j p97 blot

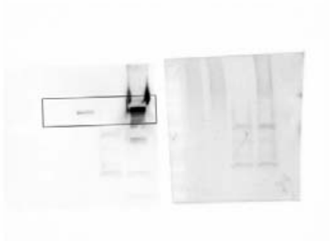

Fig. 6k CDT1 blot

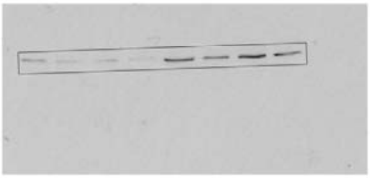

Fig. 6k PCNA blot

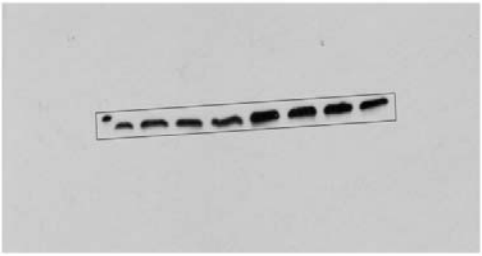

Supplementary Figure 8. Uncropped blots used to assemble main figures

Collection of uncropped western blots that are shown in the main figures. Blots are labeled according to their appearance in the main figures supplemented by the information of the detected protein(s).

RNAi#	gene	locus	predicted human ortholog	library	wt developmental phenotype	wt embryonic lethality	*cdc-48.1(tm544)* developmental phenotype	*cdc-48.1(tm544)* embryonic lethality
1	*agef-1*	*Y6B3A.1*	ARGEF1 or ARGEF2	Ahringer library	weak DD	strong	x	strong
2	*apc-11*	*F35G12.9*	APC11	ORFeome library	x	very weak	x	very weak
3	*apc-2*	*K06H7.6*	APC2	ORFeome library	x	100%	x	100%
4	*atx-3*	*F28F8.6*	ATX3	ORFeome library	x	x	x	very weak
5	*B0393.6*	*B0393.6*	unknown	Ahringer library	x	very weak	x	mild
6	*B0393.6*	*B0393.6*	unknown	ORFeome library	x	x	x	mild
7	*bir-2*	*C50B8.2*	BRIC5	Ahringer library	x	x	x	x
8	*bir-2*	*C50B8.2*	BRIC5	ORFeome library	x	x	x	x
9	*brc-1*	*C36A4.8*	BRCA1	Ahringer library	x	100%	x	100%
10	*brc-1*	*C36A4.8*	BRCA1	ORFeome library	x	x	x	very weak
11	*brc-2*	*T07E3.5*	BRCA2	Ahringer library	x	very weak	x	very weak
12	*brc-2*	*T07E3.5*	BRCA2	ORFeome library	x	x	x	x
13	*brd-1*	*K04C2.4*	BARD1	ORFeome library	x	x	x	very weak
14	*btb-13*	*ZC204.11*	KLHL35 or KLHL6	Ahringer library	x	x	x	very weak
15	*btb-13*	*ZC204.11*	KLHL35 or KLHL6	ORFeome library	x	very weak	x	x
16	*C17E4.3*	*C17E4.3*	RNF173	Ahringer library	x	x	x	very weak
17	*C17E4.3*	*C17E4.3*	RNF173	ORFeome library	x	very weak	x	very weak
18	*C34D4.14*	*C34D4.14*	TRIP12	Ahringer library	x	x	x	weak
19	*C44E4.1*	*C44E4.1*	UBR4	Ahringer library	x	x	x	very weak
20	*C53A5.6*	*C53A5.6*	unknown	Ahringer library	LL	LL	LL	LL
21	*C53A5.9*	*C53A5.9*	unknown	Ahringer library	x	very weak	x	very weak
22	*cdt-2*	*T01C3.1*	DCAF2	ORFeome library	x	very weak	x	very weak
23	*cin-4*	*ZK1127.7*	TOP2	Ahringer library	x	strong	x	100%
24	*cin-4*	*ZK1127.7*	TOP2	ORFeome library	x	n.d.	x	100%
25	*csn-1*	*Y59A8A.1*	CSN1	ORFeome library	x	100%	x	strong
26	*csn-5*	*B0547.1*	CSN5	Ahringer library	x	x	x	very weak
27	*csn-5*	*B0547.1*	CSN5	ORFeome library	x	very weak	x	weak
28	*cul-1*	*D2045.6*	CUL1	Ahringer library	x	very weak	x	mild
29	*cul-1*	*D2045.6*	CUL1	ORFeome library	x	100%	x	100%
30	*cul-2*	*ZK520.4*	CUL2	ORFeome library	x	x	x	x
31	*cul-6*	*K08E7.7*	CUL1	Ahringer library	x	x	x	x
32	*cul-6*	*K08E7.7*	CUL1	ORFeome library	x	x	x	very weak
33	*cyld-1*	*F40F12.5*	CYLD1	Ahringer library	weak DD	x	weak DD	weak
34	*cyld-1*	*F40F12.5*	CYLD1	ORFeome library	x	x	slim	weak
35	*cyn-4*	*F59E10.2*	PPIL2	Ahringer library	x	x	x	x
36	*cyn-4*	*F59E10.2*	PPIL2	ORFeome library	x	x	x	very weak
37	*D2085.4*	*D2085.4*	UBE3C	Ahringer library	x	x	x	x
38	*D2089.2*	*D2089.2*	unknown	ORFeome library	x	very weak	x	x
39	*D2089.2*	*D2089.2*	unknown	Ahringer library	x	very weak	x	very weak

40	*dcn-1*	*H38K22.2*	DCNL2	Ahringer library	x	mild	x	strong
41	*dcn-1*	*H38K22.2*	DCNL2	ORFeome library	x	strong	x	strong
42	*ddb-1*	*M18.5*	DDB1	ORFeome library	x	very weak	x	very weak
43	*dre-1*	*K04A8.6*	DRE1	ORFeome library	DD, LA	LL	DD, LA	LL
44	*dre-1*	*K04A8.6*	DRE1	ORFeome library	DD	x	DD, LA	x
45	*eel-1*	*Y67D8C.5*	HUWE1	Thermo Scientific	x	very weak	x	very weak
46	*eel-1*	*Y67D8C.5*	HUWE1	ORFeome library	x	very weak	x	x
47	*eif-3.E*	*B0511.10*	EIF3E	Ahringer library	x	x	x	very weak
48	*eif-3.E*	*B0511.10*	EIF3E	ORFeome library	DD, PV	sterile	DD, PV	sterile
49	*elb-1*	*Y41C4A.10*	ELOB	Ahringer library	x	very weak	x	very weak
50	*emb-27*	*F10B5.6*	CDC16	ORFeome library	x	100%	x	100%
51	*empty*	*empty*	x	control vector	x	x	x	x
52	*F10D7.5*	*F10D7.5*	RNF67	Ahringer library	x	x	x	x
53	*F17C11.10*	*F17C11.10*	WDHD1	Ahringer library	x	x	x	x
54	*F35F11.1*	*F35F11.1*	CDC73	ORFeome library	x	100%	DD	100%
55	*F53C11.7*	*F53C11.7*	WDR68	Ahringer library	x	very weak	x	x
56	*F55A3.3*	*F55A3.3*	SPT16 (FACT140)	Ahringer library	x	100%	LL	LL
57	*F55A3.7*	*F55A3.7*	SPT16 (FACT140)	Ahringer library	x	LA	DD, LL	sterile
58	*flh-1*	*Y11D7A.12*	unknown	ORFeome library	x	very weak	x	very weak
59	*fsn-1*	*C26E6.5*	unknown	Ahringer library	x	x	x	strong
60	*fsn-1*	*C26E6.5*	unknown	ORFeome library	x	x	x	x
61	*fzy-1*	*ZK177.6*	CDC20	Ahringer library	x	100%	x	100%
62	*fzy-1*	*ZK177.6*	CDC20	ORFeome library	x	strong	x	strong
63	*gei-17*	*W10D5.3*	PIAS1 or PIAS3	Ahringer library	x	weak	x	very weak
64	*gei-17*	*W10D5.3*	PIAS1 or PIAS3	ORFeome library	x	weak	x	weak
65	*gmn-1*	*Y75B8A.17*	Geminin	ORFeome library	x	x	x	very weak
66	*H34C03.2*	*H34C03.2*	USP11	Ahringer library	x	very weak	x	weak
67	*hmg-3*	*C32F10.5*	SSRP1 (FACT80)	ORFeome library	x	very weak	x	very weak
68	*hmg-4*	*T20B12.8*	SSRP1 (FACT80)	Ahringer library	x	x	DD, PV	x
69	*hrdl-1*	*F26E4.11*	AMFR	Ahringer library	DD	strong	x	mild
70	*hsr-9*	*T05F1.6*	unknown	Ahringer library	x	x	x	very weak
71	*hsr-9*	*T05F1.6*	unknown	ORFeome library	x	very weak	x	very weak
72	*hus-1*	*H26D21.1*	HUS1	ORFeome library	x	x	x	x
73	*ikb-1*	*C04F12.3*	BCL3	Ahringer library	x	x	x	x
74	*ikb-1*	*C04F12.3*	BCL3	ORFeome library	x	x	x	very weak
75	*mat-1*	*Y110A7A.17*	CDC27	ORFeome library	x	strong	x	100%
76	*mat-3*	*F10C5.1*	CDC23	Ahringer library	x	strong	x	strong
77	*mat-3*	*F10C5.1*	CDC23	ORFeome library	x	100%	x	100%
78	*mcm-7*	*F32D1.10*	MCM7	ORFeome library	x	100%	PV	sterile
79	*mig-32*	*F11A10.3*	RNF3A	Ahringer library	x	x	x	x
80	*mnat-1*	*F53G2.7*	MAT1	ORFeome library	x	x	x	weak
81	*mrt-2*	*Y41C4A.14*	RAD1	ORFeome library	x	very weak	x	very weak
82	*mus-101*	*F37D6.1*	TOPBP1	Ahringer library	x	very weak	x	very weak

83	*ntl-4*	*C49H3.5*	unknown	ORFeome library	x	very weak	x	x
84	*pabp-2*	*C17E4.5*	PABP2	Ahringer library	x	strong	DD	sterile
85	*pabp-2*	*C17E4.5*	PABP2	ORFeome library	x	strong	DD	100%
86	*par-2*	*F58B6.3*	unknown	Ahringer library	x	very weak	x	x
87	*pcn-1*	*W03D2.4*	PCNA	home made	x	sterile	DD, PV	sterile
88	*peb-1*	*T14F9.4*	unknown	Ahringer library	x	very weak	x	very weak
89	*pqn-5*	*C03A7.4*	unknown	ORFeome library	x	x	x	mild
90	*rad-51*	*Y43C5A.6*	RAD51	Ahringer library	x	x	x	very weak
91	*rad-51*	*Y43C5A.6*	RAD51	ORFeome library	x	weak	x	strong
92	*rbx-1*	*ZK287.5*	RBX1	ORFeome library	x	100%	x	100%
93	*rfp-1*	*R05D3.4*	BRE1	Ahringer library	x	x	x	x
94	*rfp-1*	*R05D3.4*	BRE1	ORFeome library	x	x	x	weak
95	*rnf-113*	*K01G5.1*	RNF113A or RNF113B	Ahringer library	x	strong	x	mild
96	*sel-10*	*F55B12.3*	FBXW7	Ahringer library	x	x	x	very weak
97	*sel-10*	*F55B12.3*	FBXW7	ORFeome library	x	very weak	x	very weak
98	*skr-1*	*F46A9.5*	SKP1	Ahringer library	x	very weak	x	x
99	*skr-1*	*F46A9.5*	SKP1	ORFeome library	x	mild	x	strong
100	*skr-2*	*F46A9.4*	SKP1	Ahringer library	x	x	x	very weak
101	*skr-2*	*F46A9.4*	SKP1	ORFeome library	x	very weak	n.d.	n.d.
102	*smk-1*	*F41E6.4*	SMEK1, SMEK2 or SMEK3	ORFeome library	x	very weak	x	weak
103	*T05C1.4*	*T05C1.4*	CMTA1 or CMTA2	Ahringer library	x	x	x	x
104	*T05C1.4*	*T05C1.4*	CMTA1 or CMTA2	ORFeome library	x	very weak	x	x
105	*T10F2.4*	*T10F2.4*	PRPF19	ORFeome library	DD	100%	DD	sterile
106	*T10F2.4*	*T10F2.4*	PRPF19	Ahringer library	x	100%	x	strong
107	*T19B10.6*	*T19B10.6*	DVC1/SPRTN	Ahringer library	x	x	x	x
108	*T19B10.6*	*T19B10.6*	DVC1/SPRTN	ORFeome library	x	x	x	very weak
109	*T24D1.3*	*T24D1.3*	unknown	Ahringer library	x	very weak	x	x
110	*top-2*	*K12D12.1*	TOP2	Ahringer library	x	sterile	x	sterile
111	*ubc-12*	*R09B3.4*	UBE2F	Ahringer library	x	weak	x	mild
112	*ubc-12*	*R09B3.4*	UBE2F	ORFeome library	x	mild	x	mild
113	*ubc-13*	*Y54G2A.31*	UBE2N	ORFeome library	x	very weak	x	very weak
114	*ubc-6*	*D1022.1*	UBE2J1	Ahringer library	x	x	x	very weak
115	*ubc-6*	*D1022.1*	UBE2J1	ORFeome library	x	x	x	very weak
116	*ubxn-1*	*F23C8.4*	UBXN1 (SAKS1)	Ahringer library	x	very weak	x	x
117	*ubxn-1*	*F23C8.4*	UBXN1 (SAKS1)	ORFeome library	x	very weak	x	x
118	*ubxn-2*	*Y94H6A.9*	p47	ORFeome library	x	very weak	x	very weak
119	*ubxn-3*	*F48A11.5*	FAF1	ORFeome library	x	very weak	x	100%
120	*uev-1*	*F39B2.2*	UBE2V2	Ahringer library	x	x	x	very weak
121	*ufd-2*	*T05H10.5*	UBE4B	Ahringer library	x	x	x	very weak
122	*ufd-2*	*T05H10.5*	UBE4B	ORFeome library	x	x	x	x
123	*ufd-3*	*C05C10.6*	PLAP	ORFeome library	x	x	x	very weak
124	*usp-33*	*K09A9.4*	USP20	Ahringer library	x	very weak	x	very weak
125	*usp-46*	*R10E11.3*	USP12 or USP46	Ahringer library	x	very weak	x	very weak

126	*usp-46*	*R10E11.3*	USP12 or USP46	ORFeome library	x	very weak	x	very weak
127	*usp-7*	*H19N07.2*	USP7	Ahringer library	x	mild	x	mild
128	*vab-3*	*F14F3.1*	PAX6	Ahringer library	x	very weak	x	very weak
129	*vab-3*	*F14F3.1*	PAX6	ORFeome library	x	x	x	very weak
130	*vps-11*	*R06F6.2*	VSP11 (RNF108)	Ahringer library	x	very weak	x	very weak
131	*wee-1.3*	*Y53C12A.1*	MYT1	Ahringer library	x	100%	x	100%
132	*wee-1.3*	*Y53C12A.1*	MYT1	ORFeome library	x	no eggs layed	x	1 egg layed, dead
133	*wrn-1*	*F18C5.2*	WRN (RECQL2)	Ahringer library	x	very weak	x	x
134	*wrn-1*	*F18C5.2*	WRN (RECQL2)	ORFeome library	x	very weak	x	very weak
135	*wwp-1*	*Y65B4BR.4*	ITCH, WWP1 or WWP2	ORFeome library	x	x	x	very weak
136	*Y40B1A.2*	*Y40B1A.2*	WAC	Ahringer library	x	x	x	x
137	*zhp-3*	*K02B12.8*	unknown	ORFeome library	x	very weak	x	mild

**legend**	
x	no phenotype observed
very weak	>2% embryonic lethality
weak	>25% embryonic lethality
mild	50% embryonic lethality
strong	>75% embryonic lethality
100%	100% embryonic lethality
DD	developmental delay
PV	protruding vulva
LL	larval lethality
n.d.	not determined

Supplementary Table 1. List of candidate genes for RNAi screening and results

Table lists RNAi clones that were selected from indicated libraries and sources. Developmental phenotypes and embryonic lethality are indicated for the wt and cdc-48.1(tm544) mutant. Human orthologs of respective genes are indicated when applicable.
